# The road to astaxanthin production in tomato fruit reveals plastid and metabolic adaptation resulting in an unintended high lycopene genotype with delayed over‐ripening properties

**DOI:** 10.1111/pbi.13073

**Published:** 2019-02-01

**Authors:** Eugenia M.A. Enfissi, Marilise Nogueira, Caterina D'Ambrosio, Adriana Lucia Stigliani, Giovanni Giorio, Norihiko Misawa, Paul D. Fraser

**Affiliations:** ^1^ School of Biological Sciences Royal Holloway University of London Egham Surrey UK; ^2^ Metapontum Agrobios Research Centre ALSIA Metaponto Italy; ^3^ Res Inst Bioresources & Biotechnol Ishikawa Prefectural University Nonoichi Ishikawa Japan

**Keywords:** Metabolic engineering, ketocarotenoid, *Solanum lycopersicum* (tomato), cellular adaptation, plastid, isoprenoid

## Abstract

Tomato fruit are an important nutritional component of the human diet and offer potential to act as a cell factory for speciality chemicals, which are often produced by chemical synthesis. In the present study our goal was to produce competitive levels of the high value ketocarotenoid, astaxanthin, in tomato fruit. The initial stage in this process was achieved by expressing the 4, 4′ carotenoid oxygenase (*crtW*) and 3, 3′ hydroxylase (*crtZ*) from marine bacteria in tomato under constitutive control. Characterization of this genotype showed a surprising low level production of ketocarotenoids in ripe fruit but over production of lycopene (~3.5 mg/g DW), accompanied by delayed ripening. In order to accumulate these non‐endogenous carotenoids, metabolite induced plastid differentiation was evident as well as esterification. Metabolomic and pathway based transcription studies corroborated the delayed onset of ripening. The data also revealed the importance of determining pheno/chemotype inheritance, with ketocarotenoid producing progeny displaying loss of vigour in the homozygous state but stability and robustness in the hemizygous state. To iteratively build on these data and optimize ketocarotenoid production in this genotype, a lycopene β‐cyclase was incorporated to avoid precursor limitations and a more efficient hydroxylase was introduced. These combinations resulted in the production of astaxanthin (and ketocarotenoid esters) in ripe fruit at ~3 mg/g DW. Based on previous studies, this level of product formation represents an economic competitive value in a Generally Regarded As Safe (GRAS) matrix that requires minimal downstream processing.

## Introduction

**Figure 1:**
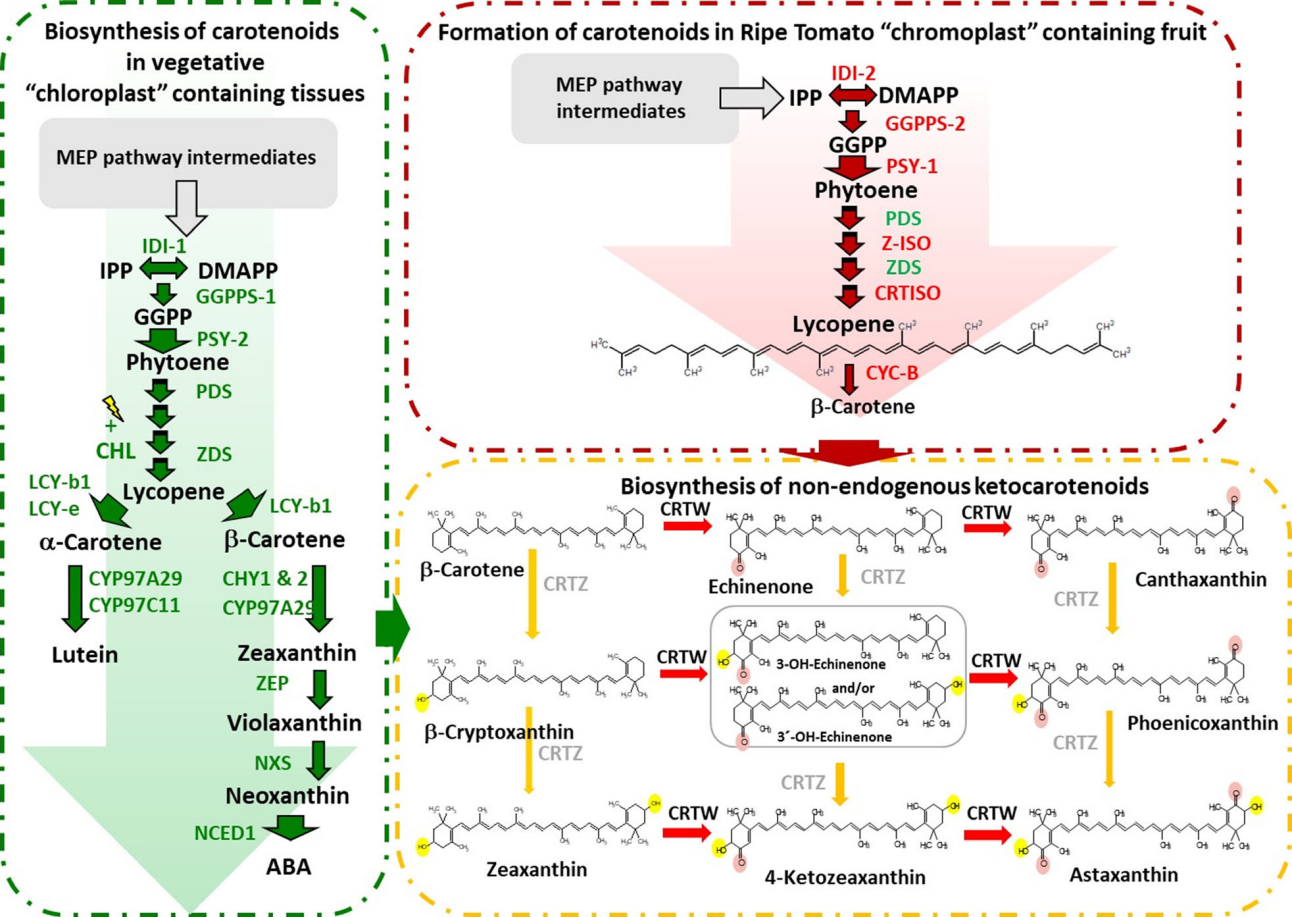
Biosynthesis of carotenoids in tomato chloroplast and chromoplast containing tissues and the conversion of endogenous metabolites to non‐endogenous ketocarotenoids. Abbreviations included for metabolites: MEP pathway‐methyl‐*D*‐erythritol 4‐phosphate pathway, IPP‐isopentenyl pyrophosphate, DMAPP‐dimethylallyl pyrophosphate, GGPP‐geranylgeranyl pyrophosphate, and ABA‐ abscisic acid. Enzymes: IDI 1 & 2, Isopentenyl diphosphate isomerase I and II; GGPPS, geranylgeranyl pyrophosphate synthases 1 & 2; PSY, phytoene synthase 1 & 2; PDS, phytoene desaturase; ZDS, ζ‐carotene desaturase; LCY‐b, Lycopene β‐cyclase; LCY‐e, lycopene ε‐cyclase; CYP97A29, P450‐type mono oxygen β ring carotenoid hydroxylase; CYP97C11, P450‐type mono oxygen ε ring carotenoid hydroxylase; CHY 1 & 2, β ring carotenoid hydroxylase; ZEP, zeaxanthin expoxidase; NXS, neoxanthin synthase; NCED1, 9‐cis‐epoxycarotenoid dioxygenase; CRTW & Z, β‐carotene 4, 4′ oxygenase and 3, 3′ hydroxylase respectively; CYC‐B, beta lycopene cyclase; CRTISO, plant carotene *cis‐trans* isomerase and Z‐ISO, 15‐*cis*‐zeta carotene isomerase.

Carotenoids represent the largest class of pigments found in nature and are present in bacteria, fungi, algae, higher plants, and animals (Britton *et al*., 2004). Industrially, they are used across multiple commercial sectors as colorants, supplements, and bioactives (Breithaupt, 2007). To date, over 700 carotenoid structures have been reported (Britton *et al*., 2004). However, just a few carotenoids, β‐carotene (provitamin A), lutein, zeaxanthin, lycopene, and the ketocarotenoids, are used commercially contributing to the $1.5 billion annual market (McWilliams, 2018). Ketocarotenoids, such as canthaxanthin and astaxanthin (Figure 1), are examples of high value pigments used in the food, feed, and health sectors (Breithaupt, 2007). They are essential to aquaculture, where their presence in feed confers intense red/pink flesh color and boosts the immune systems of farmed fish (Steven, 1948). Presently, chemical synthesis, using precursors derived from the petrochemical industry remains the most competitive production method (Ausich, 1997). However, the dwindling fossil fuel reserves and consumer demand for naturally sourced products are now driving the quest for new renewable sources of carotenoids.

Natural sources of ketocarotenoids are limited. Several ketocarotenoid forming algal species can be utilized on an industrial scale but the high production and energy intensive down‐stream processing costs incurred mean commercial products from these sources command a high price (Lorenz and Cysewski, 2000). Microbial sources are rare, such as a few marine bacteria (Misawa *et al*., 1995) and the fungus *Xanthophyllomyces dendrorhous* (Park *et al*., 2009). Whilst *In planta*, the presence of ketocarotenoids, has only been reported in the flower petals of the *Adonis* species (Cunningham and Gantt, 2005), which is not amenable to agricultural production. Although these natural sources do not represent viable industrial production platforms in their own right, biosynthetic genes isolated from them have been exploited in the metabolic engineering of heterologous hosts and the production of ketocarotenoids in crop plants has been reported on numerous occasions previously (Mortimer *et al*., 2016, 2017; Zhu *et al*., 2009). More recently the technical, production, and economic feasibility of engineered tomato fruit producing ketocarotenoids has been demonstrated as a new renewable feedstock for aquaculture (Nogueira *et al*., 2017).

Biosynthetically, ketocarotenoids are formed from β‐carotene by the action of a 4, 4′ carotenoid oxygenase (*crtW*), (Choi *et al*., 2005, 2007). CRTW is capable of introducing keto groups at the 4 and 4′ positions of the carotenoid β‐ionone rings, in the absence or presence of hydroxylation at the 3, 3′ position (Figure 1). Thus, the 4, 4′ carotenoid oxygenase can act directly on endogenous plastid derived β‐carotene or zeaxanthin as precursors. The carotenoid hydroxylase(s) from microbial (CRTZ) and plant sources can catalyze the incorporation of hydroxyl moieties at the 3, 3′ positions on the β‐ionone rings as well as act on previously ketolated β‐ionone ring carotenoids at the 4, 4′ positions (Fraser *et al*., 1998). The potential products from the actions of the hydroxylase and oxygenase on endogenous carotenoids are illustrated in Figure 1.

In this present study, an iterative journey to the optimal combination of biosynthetic gene products for astaxanthin formation in tomato fruit is described. In addition, the effects of the perturbation of the plant's metabolism on fundamental processes associated with fruit ripening, plastid differentiation and plant metabolism have been investigated.

## Results

### Generation of transgenic tomato plants producing ketocarotenoids

*Agrobacterium*‐mediated transformation was used to introduce the *crt*Z and *crt*W genes from *Brevundimonas* sp. (Mortimer *et al*., 2017) into Money Maker (MM) tomato plants. Twelve kanamycin resistant Money Maker (MM) plants were selected and the presence of the *crt*Z and *crt*W genes in the independent lines, designated MM:*Crt*ZW1 to 12, confirmed by PCR. Quantitative polymerase chain reaction (QPCR) and Southern blotting identified the presence of two or more inserts in all lines except in one single insert line. In comparison to the wild type (MM), vegetative tissues were brown in color in all transformants, apart from MM:*Crt*ZW6, which possessed a wild type phenotype. The flowers and fruit of all 12 transformants showed no observable phenotypic differences compared to MM. Leaf material from the transformants was screened by TLC for the presence of unique ketocarotenoids. All PCR positive plants with a brown phenotype showed the presence of ketocarotenoids (Figure S1). HPLC analysis was used to validate and quantify the presence of the endogenous (Table S1a) and newly formed carotenoids (Table S1b) present in vegetative material. All T0 transformants with brown leaf phenotypes had a reduced endogenous carotenoid content (Table S1a). Lutein was reduced by 82%, β‐carotene by 65% and β‐derived xanthophylls were below the detectable threshold. Reductions in chlorophylls were less pronounced although chlorophyll a was reduced by ~20%. Total ketocarotenoid content ranged from 397 μg/g DW to 473 μg/g DW, canthaxanthin being the predominant product, but hydroxylated ketocarotenoids were also present (Table S1b). Ketocarotenoids were detectable in trace amounts (<1 μg/g DW) by HPLC in the fruit (mature green and ripe) from the T_0_ plants.

A T1 generation of MM:*Crt*ZW plants were sown from the single insert line, MM:*Crt*ZW10, and the double insert primary line with the highest leaf ketocarotenoid contents, MM:*Crt*ZW12. Among the 20 progeny generated per line, phenotypic segregation was evident. Observable phenotypic differences included leaf coloration, and reduced height. QPCR and Southern blotting were used to determine the zygosity of the progeny and correlations between zygosity, seedling height at 6 weeks post germination and leaf color were made. The single insert hemizygous lines had visibly brown leaves and were reduced in height by ~50% (*P* < 0.001) compared to MM. Single insert homozygous lines had brown or pale leaves and displayed poor vigour resulting in a reduction in height of 85% (*P* < 0.001) compared to MM. The azygous lines showed no phenotypical differences compared to MM. Progeny derived from the double insert primary line showed greater phenotypic variation. Despite this variation, three azygous and three single locus hemizygous plants were identified, with phenotypes equivalent to those recorded for the single insert line. Progeny that had inherited more than one copy of the transgenes, either homozygous at one locus or hemizygous/homozygous for the two loci showed, on average, an 83% (*P* < 0.001) reduction in height, poor vigour, and a range of leaf colors from green to brown. Those hemizygous seedlings initially reduced in height, were indistinguishable in stature, from their azygous or wild type comparators when mature.

### Biochemical, cellular, and molecular characterization of MM:*CrtZW* T1 and T2 plants

#### Carotenoid contents of T1 and T2 MM:CrtZW progeny

Multi‐platform chromatographic separations using TLC, open column, UPLC and HPLC combined with UV/Vis detection were carried out to ascertain their physico‐chemical properties of the unique keto/hydroxyl carotenoids compared to authentic standards. Criteria for identification have been summarized in Table S2 and further details were presented in Mortimer *et al*. (2016, 2017), Nogueira *et al*. (2017).

Analysis of leaf tissue from hemizygous MM:*Crt*ZW T1 progeny with MM and their true azygous controls reinforced the carotenoid profiles determined in the primary transformants (Table S3a,b). Lutein content was reduced (88%) compared to the mean content in azygous plants, as was β‐carotene (60%) and all β‐ionone ring derived xanthophylls (violaxanthin and neoxanthin; below the threshold of detection). The reduction in chlorophyll a and b was again observed. Astaxanthin was detected (13–19 μg/g DW) along with other hydroxylated and ketolated carotenoids, such as phoenicoxanthin (5.6–7.3 μg/g DW) and 3′ OH‐echinenone (42–64 μg/g DW). Mono and bi ketolated carotenoids echinenone (trace) and canthaxanthin, respectively, were present. The latter being the most abundant ketolated carotenoid product (407–648 μg/g DW). The effect of the altered carotenoid and chlorophyll contents on photosynthetic capacity of the lines expressing *crt*Z and *crt*W was assessed. The maximum photochemical efficiency of photosystem II, in the dark adapted state (*F*_v_ */F*_m_), was reduced by an average of 12% (*P* < 0.001) in the hemizygous lines compared to MM or azygous lines.

Upon fruit set and development, a clear observable difference arose in the coloration of the fruit from MM:*Crt*ZW T1 progeny in the hemizygous state compared to the fruit of the MM:*Crt*ZW primary transformants, the MM and azygous controls. The fruit displayed a uniform pink phenotype, turning deep red as ripening progressed. Analysis of pigments at the mature green stage (Table S3c,d) revealed that chlorophylls a and b were reduced in all hemizygous lines compared to azygous by 71% and 55%, respectively. The endogenous carotenoids, β‐carotene, and lutein, were below the detection threshold, whilst the ketocarotenoids echinenone, canthaxanthin, phoenicoxanthin, and astaxanthin were all present at levels below 10 μg/g DW. In addition to free carotenoids, phoenicoxanthin esters were present (trace amounts <1 μg/g DW). Collectively, the total pigment content of the MM:*Crt*ZW mature green fruit were significantly reduced by >65% compared to both MM and the azygous controls. The carotenoid profile of mature green fruit from azygous plants was similar to the MM background, with typical carotenoids characteristic of chloroplast containing tissues prevailing. Likewise, mature green fruit from homozygous T1 lines maintained a carotenoid profile similar to that of the MM background and ketocarotenoids were not detected in these tissues.

The deep red coloration observed in the ripe fruit from the T1 generation of MM:*Crt*ZW plants was not due to the presence of ketocarotenoid accumulation (Table S3e,f). Unique ketocarotenoids were present in ripe MM:*Crt*ZW fruit compared to MM and the azygous controls but at low levels. For example, echinenone, canthaxanthin, phoenicoxanthin, and astaxanthin were present, up to ~25 μg/g DW, along with phoenicoxanthin esters with C14 and C16 fatty acid derivatives. The increased red color intensity of the MM:*Crt*ZW ripe fruit was due to increased lycopene content. The levels were up to 3.5‐fold higher than that found in the wild type. Concurrent with this increase in lycopene, was an increase in phytoene (2fold) and a reduction in β‐carotene to only 20–25% of the levels found in the varietal and azygous controls.

**Figure 2:**
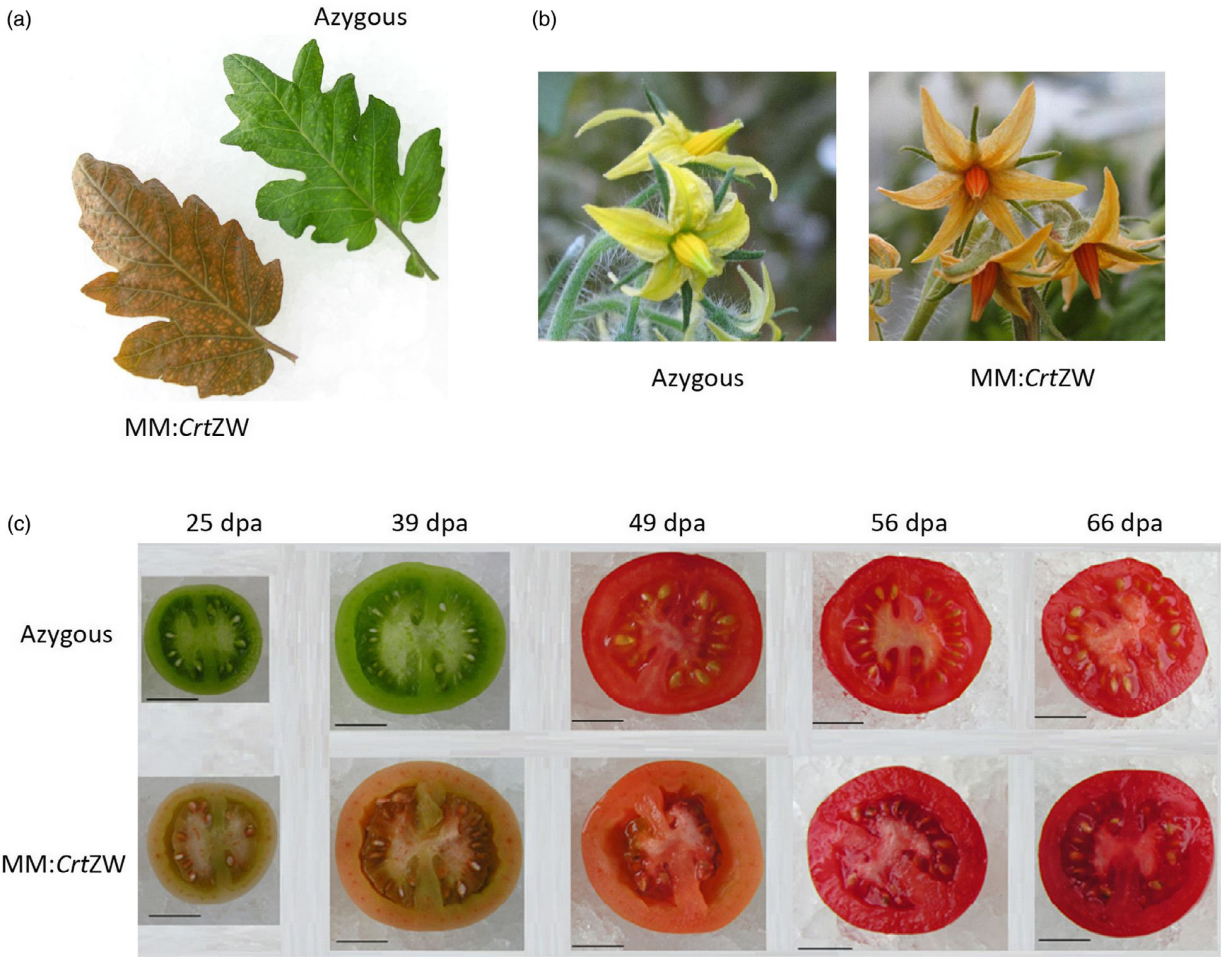
Phenotype of MM:*Crt* ZW T2 generation compared to azygous control. (a) Leaf; (b) Flowers; (c) Fruit at five stages of development from immature green (25 dpa) to ripe fruit (66 dpa). Scale bar represents 1 cm.

In order to test the inheritance of the MM:*Crt*ZW chemo/phenotype, a T2 population from the two highest astaxanthin‐producing, single insert, hemizygous T1 lines, MM:*Crt*ZW10‐16 and MM:*Crt*ZW12‐4, was generated. Segregation could be observed phenotypically, with hemizygous plants displaying a brown phenotype, homozygous a pale brown/green and dwarf phenotype and azygous plants a wild‐type green phenotype. Zygosity was checked with Southern blotting and/or QPCR. Measurements of photosynthetic capacity and analysis of carotenoid and chlorophyll content of the leaf tissue (Table S4a,b) confirmed the results obtained in the T1 generation. Fruit chemo (Table S4c) and phenotypes (Figure 2) confirmed that previously found in the T1 generation. In order to ascertain the stability of the phenotype under different environmental conditions, an additional T2 crop was grown under glasshouse conditions in a second geographical location (Metaponto, Italy) and the carotenoid contents of leaf and ripe fruit were analysed by HPLC. The carotenoid profile of both the leaf and ripe fruit material confirmed the formation of ketocarotenoids and quantitative changes in endogenous carotenoids (Table 1).

**Table 1:** Pigments in leaf tissue and ripe fruit from MM:*Crt*ZW lines grown in a second location and hybrids with MM:*Crt*ZW and lines expressing either an additional lycopene cyclase, (HC/MM:*Crt*ZW) or an additional lycopene cyclase and an additional β‐carotene hydroxylase (HU/MM:*Crt*ZW)

μg/g DW	Leaf			Ripe fruit		
	MM:*Crt*ZW	HC/MM:*Crt*ZW	HU/MM:*Crt*ZW	MM:*Crt*ZW	HC/MM:*Crt*ZW	HU/MM:*Crt*ZW
Lycopene	n/d	n/d	n/d	1126.8 ± 109.9	**17.9 ± 1.5** ***	**10.5 ± 2** ***
β‐Carotene	100.8 ± 9.8	**146.1 ± 17** ***	**251.6 ± 10.6** ***	12.8 ± 3.8	**269.5 ± 47.7** **	**80.6 ± 23** *
Lutein	342 ± 30.5	**108.3 ± 11.8** ***	**97.4 ± 15.8** ***	n/d	n/d	n/d
Violaxanthin	6.2 ± 10.8	6.4 ± 8.6	**432.9 ± 38.6** ***	n/d	n/d	16.3 ± 7.1
Echinenone	278 ± 42.6	**315.3 ± 19.4** *	**181.8 ± 2.7** *	n/d	131.7 ± 15.4	154.7 ± 16
3′‐OH‐Echinenone	113.8 ± 26.2	101.2 ± 15.9	**174.5 ± 1.5** **	n/d	37.0 ± 4.8	37.5 ± 5.2
Canthaxanthin	634.1 ± 82.8	**887 ± 95.7** ***	n/d	19.8 ± 4.8	**611.5 ± 41.1** ***	59 ± 35.1
Phoenicoxanthin	662.4 ± 77	**889.6 ± 70.6** ***	**74.3 ± 6.8** ***	36.7 ± 8.6	**481.9 ± 29.3** ***	98.8 ± 42.6
Adonixanthin	372.8 ± 46.8	**184.1 ± 14.6** ***	**118.6 ± 5.5** ***	n/d	14.6 ± 2	26.2 ± 2.6
Astaxanthin	298.4 ± 66.1	276.6 ± 28.9	362.5 ± 20.1	38.7 ± 5.5	**140.8 ± 6.9** ***	**179.4 ± 15** ***
Keto‐antheraxanthin	426.3 ± 155.7	**230.2 ± 105.7** **	**4740.3 ± 75.8** ***	n/d	43.8 ± 6.3	139.6 ± 25.9
Keto‐esters	n/d	n/d	n/d	71.0 ± 6.9	**1390.6 ± 96.5** ***	**1532.5 ± 97.3** ***
Unidentified ketos	157.9 ± 40.2	153.6 ± 29.8	**414.4 ± 10.2** ***	n/d	n/d	n/d
Total keto	2943.7 ± 395.1	3037.5 ± 154.2	**6796.3 ± 28.2** ***	166.3 ± 49.4	**2847.8 ± 347.2** ***	**2227.6 ± 389.9** ***
Total carotenoids	3392.6 ± 418.5	3298.2 ± 159.4	**6848.2 ± 89.4** ***	1305.9 ± 268.1	**3135.2 ± 417.2** ***	**2332.2 ± 435.5** **
Chlorophyll a	10184.2 ± 1131.7	10798.8 ± 492.7	**11955.4 ± 79.9** ***	n/d	n/d	n/d
Chlorophyll b	4786.7 ± 664.2	4319.9 ± 318.2	**8091 ± 192.7** ***	n/d	n/d	n/d

The values shown are the mean ± standard deviation, where *n* = 3–10. Significant differences, shown in bold, between the mean of MM:*Crt*ZW and those of HC/MM:*Crt*ZW and HU/MM:*Crt*ZW for each compound within the same tissue were evaluated using the Student's *t*‐tests (*P < 0.05; **P < 0.01; ***P < 0.001). n/d: Not detected.

In order to ascertain if color and ripening had been uncoupled in the MM:*Crt*ZW genotypes, analysis of the T2 generation throughout fruit development was carried out. Fruits were harvested from hemizygous and azygous plants of line MM:*Crt*ZW10‐16 (the highest astaxanthin producing line) at designated time points post anthesis, the staging being 25 days post anthesis (dpa), 39, 49, 56, and 66 dpa. Pigment levels (Table S4c) along with fruit physiology and ripening parameters were determined. Figure 2c shows the comparative fruit phenotypes. Although the MM:*Crt*ZW have a pink coloration compared to their green fruited controls, fruit firmness, and the duration from anthesis suggest that the MM:*Crt*ZW develop at the same rate to the mature green stage. Then, in comparison to the azygous, took approx. 10 days longer to develop color and approx. 16 days longer to soften, with the azgyous achieving a fruit firmness percentage of 66% at 49 dpa whilst MM:*Crt*ZW achieved the same fruit firmness at 66 dpa (Table S5). At 66 dpa, when the MM:*Crt*ZW fruits had developed a deep red color, ketocarotenoids accumulated to 100 μg/g DW, astaxanthin representing the majority of the ketocarotenoid (75 μg/g DW). Lycopene levels were almost twofold higher than the control and β‐carotene in the MM:*Crt*ZW was reduced by 60%. Fruit were no different in circumference or weight compared to wild type.

#### Expression of carotenoid biosynthetic genes in response to ketocarotenoid production

Transcript levels of carotenoid pathway genes were quantified in T2 lines at three stages of fruit development and ripening, 39, 49, and 56 dpa using qRTPCR. At 39 dpa, when the fruit had reached mature green stage, the only statistically significant changes in transcript content detected were in the ripening related lycopene β‐cyclase, *CYC*B, and the chloroplast lycopene β‐cyclase, *LCY*B, which were both down‐regulated by approximately 40% (Table 2). However, at 49 dpa all transcripts quantified, except zeaxanthin epoxidase, ZEP, were significantly different in MM:*Crt*ZW fruit compared to the azygous control (Table 2). The majority of the genes were down‐regulated. In the case of early pathway genes, 1‐deoxy‐D‐xylulose‐5‐phosphate synthase, *DXS,* was down‐regulated by approximately 85% and the phytoene synthases, *PSY‐*1 and *PSY*‐2, were down‐regulated by over 99% compared to the control. Transcripts of genes relating to carotenoid desaturation and isomerization steps were less severely affected but were still reduced by over 50%. In the case of cyclization, *LCY*B transcript levels were higher than in the control whilst the *CYC*B transcripts were reduced compared to the control. This may reflect a delay in ripening and the absence of substrate for the CYCB. At 56 dpa most genes in the pathway were significantly up‐regulated in lines expressing *crt*Z and *crt*W relative to the control with early pathway genes such as *DXS* and *PSY‐*1/*PSY*‐2 and the β‐carotene hydroxylase, *CRTRB‐1*, showing the largest increases.

**Table 2:** Transcript levels of carotenoid pathway genes in hemizygous MM:*Crt*ZW T2 fruit at 39, 49, and 56 dpa relative to the azygous control

Gene	Transcript level relative to azygous control		
	39 dpa	49 dpa	56 dpa
*DXS*	1.52 ± 0.13	**0.26 ± 0.03** ***	**4.15 ± 0.42** ***
*GGPPS1*	ND	**1.72 ± 0.01** **	1.37 ± 0.33
*GGPPS2*	ND	**0.02 ± 0.005** *	1.68 ± 0.03
*PSY2*	1.02 ± 0.29	**0.07 ± 0.02** *	**4.57 ± 1.05** **
*PSY1*	0.79 ± 0.07	**0.01 ± 0.002** **	**2.50 ± 0.43** **
*PDS*	0.95 ± 0.11	**0.52 ± 0.06** **	**1.68 ± 0.10** ***
*ZDS*	1.59 ± 0.35	**0.30 ± 0.05** ***	**1.33 ± 0.18** *
*CRTISO*	ND	**0.36 ± 0.01** ***	**1.56 ± 0.24** **
*ZISO*	ND	**0.039 ± 0.01** ***	**2.95 ± 0.19** ***
*LCYB*	**0.61 ± 0.03** *	**3.33 ± 0.40** ***	**1.60 ± 0.20** **
*CYCB*	**0.63 ± 0.11** **	**0.65 ± 0.07** **	**0.65 ± 0.29** *
*LCYE*	0.76 ± 0.02	**20.49 ± 2.2** ***	**1.17 ± 0.10** *
*CRTRB1*	ND	**0.20 ± 0.03** ***	**3.15 ± 0.08** ***
*ZEP*	1.06 ± 0.30	1.05 ± 0.38	0.70 ± 0.21
*VDE*	1.26 ± 0.06	**2.49 ± 0.23** ***	**6.78 ± 0.32** ***

Total RNA was extracted from a minimum of three pooled fruit from three plants per stage. qRT‐PCR was performed with gene‐specific primers for *DXS*, 1‐deoxy‐D‐xylulose‐5‐phosphate synthase; *GGPPS1*, geranylgeranyl pyrophosphate synthase‐1; *GGPPS2*, geranylgeranyl pyrophosphate synthase‐2; *PSY1*, phytoene synthase‐1; *PSY2*, phytoene synthase‐2; *PDS*, phytoene desaturase; *ZDS*, ζ‐carotene desaturase; *CRTISO*, carotene isomerise; *crtI*, bacterial phytoene desaturase; *LCYB*, β‐lycopene cyclase; *CYCB*, lycopene cyclase B; *LCYE*, ε‐lycopene cyclase. Expression data was normalized to the expression level of actin. Statistical determinations are shown as mean ± standard deviation, where *n* = 3–6. Significant differences are shown in bold. The P‐Values, as determined by students *t* ‐tests, are indicated as follows: *0.05, **0.01, ***0.001. ND, not determined.

#### Ultrastructure changes to plastids

**Figure 3:**
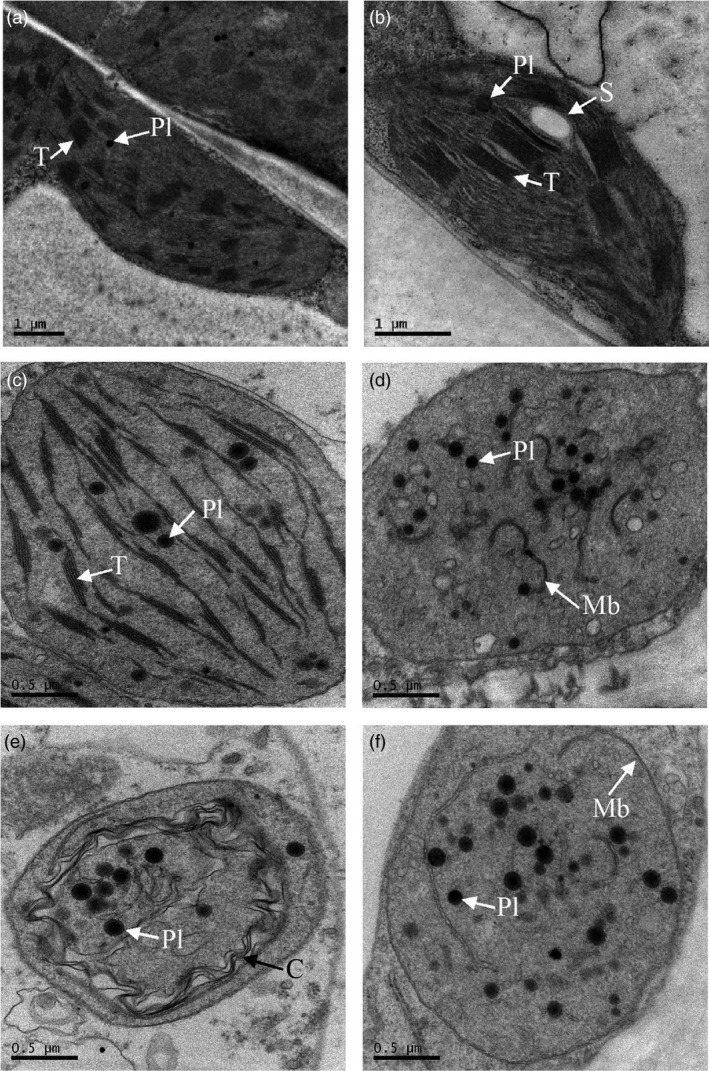
Transmission electron micrographs of plastids from azygous control and MM:*Crt* ZW leaf and fruit pericarp. Panels (a) and (b) leaves from azygous control and MM:*Crt* ZW, respectively. Panels (c) and (d) mature green pericarp tissue from azygous control and MM:*Crt* ZW, respectively. Panels (e) and (f) ripe pericarp tissue from azygous control and MM:*Crt* ZW, respectively. C, lycopene crystals; Mb, membranous structures; Pl, plastoglobule; S, starch granule; T, thylakoid membranes.

Transmission electron microscopy was performed on leaf, mature green, and ripe fruit tissues (Figure 3). Chloroplasts visualized in leaf tissues from the MM:*Crt*ZW lines and azygous controls were similar, with clear defined thylakoid membrane stacks, grana and densely staining plastoglobuli. Azygous lines at the mature green fruit stage also contained these typical chloroplast structures. However, the MM:*Crt*ZW mature green fruit contained chloroplasts lacking defined thylakoid stacks and instead contained disordered membrane fragments and plastoglobuli. Comparison of MM:*Crt*ZW and azygous control chromoplasts present in ripe fruit were similar, although the MM:*Crt*ZW genotypes appeared to contain fewer membrane structures and more, smaller, denser plastoglobuli than the controls.

**Figure 4:**
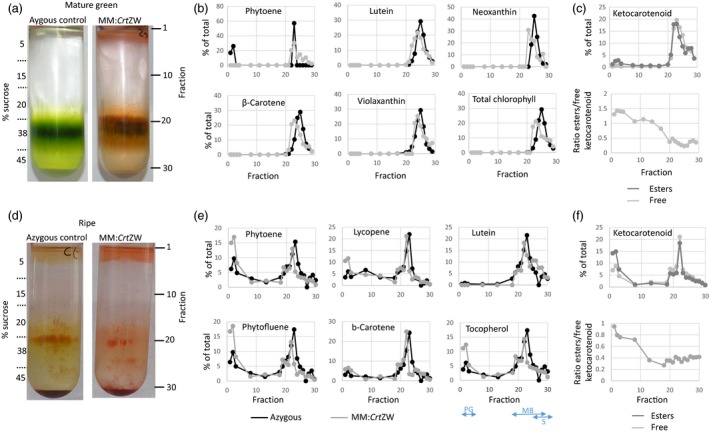
Subplastidial carotenoid sequestration in response to non‐endogenous carotenoid synthesis. Panels (a) and (d). Fractionation of subplastidial components of chloroplasts from mature green fruit and chromoplasts from ripe fruit, respectively, from azygous control and MM:*Crt* ZW lines. Panels (b) and (e) show the distribution of endogenous carotenoid contents in the isolated fractions from mature green and ripe fruit, respectively, in azygous control and MM:*Crt* ZW lines. Panels (c) and (f) show the distribution of free and esterified ketocarotenoids in mature green and ripe MM:*Crt* ZW fruit, respectively. Two‐headed arrows labelled PG, MB and S indicate the approximate fraction range distribution of plastoglobules, membranes and stroma, respectively.

To investigate the sub‐plastidial sequestration of novel ketocarotenoids, sucrose density gradient centrifugation as described in Nogueira *et al*. (2013) was carried out. Using the Plastoglobulin 35, PSBA (for photosystem II protein D1), TIC40 (for translocon at the inner envelope of chloroplasts), TOC75 (for translocon at the outer envelope of the chloroplasts), and the stromal RBCL (for ribulose‐1,5‐bisphosphate carboxylase/oxygenase large subunit) to identify the fractions. The distribution of pigments in azygous control mature green fruit, indicated that the chlorophylls and carotenoids were, as expected, closely associated with the membrane fractions (fractions 18–28, Figure 4a–c). This was also true of the low levels of endogenous carotenoids and chlorophylls detected in the fractions from the mature green fruit of the MM:*Crt*ZW line. Ketocarotenoids, which accounted for over 40% of the isoprenoids detected, were also associated with the membrane fractions and showed the same distribution pattern as the endogenous carotenoids. However, ketocarotenoid esters were found to be preferentially associated with the plastoglobuli fractions (fractions 1–3). In fractions from ripe fruit of the azygous control lines the pigments were, again, predominantly associated with the membrane fractions, although they were also detected in the pastoglobuli where phytoene and phytofluene, in particular, accumulated. In the MM:*Crt*ZW chromoplast fractions distribution of carotenoids, ketocarotenoids, and tocopherols was similar to the major pigments in the azygous control (Figure 4d–f) with the majority being associated with the membrane fractions. However, the percentage of phytoene, phytofluene, lycopene, and tocopherol associated with the plastoglobuli fractions (fractions 1–3) was 2–3 fold higher in the MM:*Crt*ZW lines compared to the control. Similarly to the mature green result, keto‐esters also preferentially accumulated in the plastoglobuli.

#### Changes in intermediary metabolism associated with altered carotenoids

To assess the plasticity in intermediary metabolism in response to novel ketocarotenoid formation GC‐MS based metabolite profiling was carried out. Principal Component Analysis (PCA) was performed on all datasets from leaf, mature green (39 dpa), and ripe tissues from the azygous control and MM:*Crt*ZW hemizygous genotype (Figure S2). Ripe was defined as red ripe which was designated as 49 and 66 dpa for the azygous control and MM:*Crt*ZW, respectively based on colour development and a fruit firmness of <70%. Cluster analysis gave rise to three distinctive entities corresponding to leaf, mature green, and ripe tissues. Subsequent genotypic separation was evident when independent PCA was carried out with a single tissue type from azygous and MM:*Crt*ZW lines. The overall compositional variation explained by the metabolites analysed for both the leaf and mature green material between MM and MM:*Crt*ZW was low (e.g. 18% component 1). However, the ripe tissues from the two genotypes showed greater variation in chemical composition (up to 40% component 1).

Comparisons between individual metabolites revealed that significant changes had occurred across multiple sectors of intermediary metabolism (Table 3). Of the amino acids quantified, over 50% were elevated in both the mature green and ripe tissue of the MM:*Crt*ZW line with the increases being greater at ripe stage. The significant increases in γ‐amino‐butyric acid, glutamic acid, and pyroglutamic acid correlated biosynthetically with the dramatic increases in the polyamine putrescine. Given the dramatic membrane associated ultrastructural changes, comparatively major fatty acid levels did not alter. In contrast, long chain hydrocarbons and amyrins associated with the cuticle displayed an increased trend especially in the MM:*Crt*ZW ripe fruit. Besides carotenoids, related isoprenoids such as tocopherols and phytosterols were increased in ripe tissue of the MM:*Crt*ZW. In contrast, phytol was reduced in the leaf material and a similar trend shown in mature green MM:*Crt*ZW which correlated with lower chlorophyll levels. The organic acid content was altered significantly in both mature green and ripe tissues of the MM:*Crt*ZW genotype compared to the azygous control. Increases in glycolytic intermediates were most pronounced in the MM:*Crt*ZW ripe tissue. Sugars derived from cell wall remodelling e.g. arabinose, mannose, galacturonic acid, and gluconic acid were reduced significantly or displayed a reduced trend in MM:*Crt*ZW ripe fruit. Sucrose was elevated at both the mature green and ripe stages in the MM:*Crt*ZW genotype, while glucose and fructose were both reduced. In order to ascertain the possibility that the changes arising in the ripe fruit were solely due to altered ripening the ratio of metabolite present in the ripe to mature green tissue was calculated for the MM:*Crt*ZW and its azygous control. In this case 50% of the metabolites displayed the same trend in terms of decreased or increased levels upon the transition from mature green to ripe. Trends in individual metabolites present in the leaf material showed little correlation with metabolite changes experienced in mature green or ripe tissues of the MM:*Crt*ZW. The most notable similarity being α‐tocopherol increased while phytol and chlorophyll were reduced in both leaf and fruit of MM:*Crt*ZW.

**Table 3:** Metabolite levels in polar extracts (intermediary metabolism) from leaf, mature green, and ripe fruit tissues of MM:*Crt*ZW plants relative to the azygous control

	Leaf	Mature green fruit	Ripe fruit
Amino acid			
Alanine	n/d	n/d	**100** ***
Aspartic acid	1.31	**2.83** ***	**5.96** **
γ‐Aminobutyric acid	**0.15** *	**1.43** *	**9.08** ***
Glutamic acid	n/d	n/d	**100** **
Glutamine	n/d	**100** ***	n/d
Pyroglutamic acid	1.18	1.99	**2.41** ***
Serine	**0.23** ***	**1.66** ***	**2.16** **
Threonine	**0** *	2.40	**100** **
Tyrosine	**0** ***	n/d	n/d
Valine	**0.31** ***	**100** **	n/d
Fatty acid			
C16:0	1.00	0.99	1.07
C17:0	**0.52** **	**1.82** **	1.21
C18:0	1.12	0.83	0.92
C18:0 ME	1.51	0.91	0.84
C18:1	n/d	0	1.18
C18:1 cis9 ME	n/d	n/d	**0** *
C18:2 trans9,12 ME	1.25	1.42	1.09
C20:0	**0** **	0.40	n/d
C22:0 ME	**1.57** *	n/d	n/d
C23:0	**100** **	n/d	n/d
C24:0	1.30	**100** *	1.04
C26:0	1.69	0.96	0.78
Hydrocarbon			
C29H60	2.02	100	3.52
C30H62	2.13	n/d	5.64
C31H64	1.79	0.89	1.21
C32H66	1.74	n/d	100
C33H68	2.55	n/d	**100** *
Isoprenoid			
α‐Tocopherol	1.51	1.90	**1.62** **
γ‐Tocopherol	0.74	**100** **	**0.16** **
α‐Tocopherolhydroquinone	n/d	**1.61** **	**0** *
α‐Amyrin	n/d	1.04	1.20
β‐Sitosterol	0.99	1.21	**1.83** **
Phytol	**0.22** ***	0.61	n/d
Organic acid			
Aconitic acid	n/d	n/d	**100** **
Ascorbic acid	n/d	n/d	100
Citraconic acid	n/d	**0** **	0.88
Citramalic acid	1.22	0.98	100
Citric acid	1.25	0.91	**1.85** *
Fumaric acid	0.65	**3.04** **	**2.71** ***
Isocitric acid	**100** **	n/d	n/d
Itaconic acid	n/d	**1.79** *	**3.21** ***
Lactic acid	2.08	0.59	0.60
Maleic acid	**0.46** **	**2.01** *	**100** **
Malic acid	1.28	**1.59** ***	**1.58** **
Succinic acid	1.13	n/d	1.04
Phenylpropanoid			
trans‐Caffeic acid	1.27	n/d	n/d
Phosphate			
Methylphosphate	1.29	**4.55** *	**100** **
Phosphate	1.43	0.77	**2.16** **
Polyamine			
Putrescine	n/d	**100** *	**100** **
Sugar			
Arabinose	**1.24** ***	n/d	**0** ***
Disaccharide 47.6 min	0.95	n/d	n/d
Disaccharide 50.7 min	**0.47** ***	n/d	n/d
Erythronic acid	**1.92** **	n/d	100
Fructose	**0.36** **	0.70	0.60
Galactose	**87.65** **	0.89	**100** *
Galacturonic acid	1.02	n/d	0.78
Glucaric acid	0.92	1.11	**100** **
Gluconic acid	0.39	**1.64** ***	0.87
Glucose	**0.46** **	0.71	0.52
Glyceric acid	**0.46** **	0.82	0.90
Glycerol	**0.32** **	0.76	0.57
Glycerol‐3‐phosphate	0.66	0.81	**3.14** **
Inositol	**0.35** ***	**1.20** **	**1.20** *
Maltose	**0.43** ***	n/d	n/d
Mannose	**0.46** **	0.71	0.52
Sedoheptulose	**2.61** **	n/d	1.67
Sucrose	0.91	**1.28** ***	**1.36** **
Trehalose	2.53	n/d	n/d

Ratios were calculated from the mean values of a minimum of three biological and three technical replicates. A value of 0 indicates an infinite decrease, whilst a value of 100 indicates an infinite increase in MM:*Crt*ZW samples relative to the control. n/d = Not detected in either sample. Significant differences are indicated in bold and were determined by Student's *t*‐tests. The *P*‐Values are indicated as follows: *0.05, **0.01, ***0.01.

### Phenotype stability and further engineering of the carotenoid pathway in tomato lines expressing a β‐carotene ketolase and β‐carotene hydroxylase from *Brevundimonas* sp

The reduction in β‐carotene levels in MM:*Crt*ZW was a key observation across multiple generations and was independent of the environments tested. Therefore, a MM:*Crt*ZW line was crossed with a tomato line over expressing the tomato *LCYB* under the constitutive cauliflower mosaic virus 35S promoter. This line has previously been shown to convert almost all the lycopene present in fruit to β‐carotene (D'Ambrosio *et al*., 2004) and, as shown previously, (Nogueira *et al*., 2017), the presence of an additional cyclase leads to an increase in the pool of β‐carotene available for metabolism to ketocarotenoids. These progeny from the genetic cross were designated HC/MM:*Crt*ZW, and analyses of ripe fruit from the F1 generation contained significantly higher levels of ketocarotenoids compared to MM:*Crt*ZW parental line (Table 1). Ketocarotenoids in HC/MM:*Crt*ZW lines accounted for 90% of the total carotenoid content of ripe fruit with 50% of those found to be in esterified forms. De‐esterification of the extracts with cholesterol esterase revealed the majority of the esters (70%) to be phoenicoxanthin rather than astaxanthin (Figure S3). Lycopene in these fruit was reduced to <20 μg/g DW but β‐carotene content was the same as in ripe MM wild type fruit (269 μg/g DW). Accumulation of mono‐hydroxylated but biketolated compounds, such as phoenicoxanthin and canthaxanthin, in the HC/MM:*Crt*ZW lines indicated that hydroxylation was a limiting step in the formation of the target end product astaxanthin. Therefore, in order to enhance the formation of the end product astaxanthin, the carotenoid pathway was further engineered by the introduction of an additional tomato β‐carotene hydroxylase (*CRTRB‐2*) under the control of the 35S promoter. The lines used to generate the hybrid had been characterized previously and reported to accumulate increased levels of xanthophylls in both leaf and fruit tissues (D'Ambrosio *et al*., 2011). Tomato genotypes, HU/MM:*Crt*ZW, expressing the full combination of genes were analysed. Leaf and ripe fruit of the resulting HU/MM:*Crt*ZW lines were found to contain elevated levels of astaxanthin relative to the MM:*Crt*ZW and HC/MM:*Crt*ZW lines with free astaxanthin reaching 362 μg/g DW in leaf and 179 μg/g DW in ripe fruit (Table 1, Figure S4). Additionally, unlike the HC/MM:*Crt*ZW lines, where the majority of keto‐esters were phoenicoxanthin, in the HU/MM:*Crt*ZW fruit the majority of esters were found to be astaxanthin (73%), with phoenicoxanthin accounting for just 20% of the esters (Figure S3). Lycopene levels in ripe fruit were significantly reduced to ~10 μg/g DW and β‐carotene was also reduced (81 μg/g DW). In addition to ketocarotenoids, violaxanthin was also detectable as a novel product in ripe fruits of HU/MM:*Crt*ZW. Overall, in the HU/MM:*Crt*ZW plants, total ketocarotenoids were increased twofold in leaf to >6.8 mg/g DW and over 13‐fold in ripe fruit to >2 mg/g DW compared to MM:*Crt*ZW.

## Discussion

### Tomato as a cell factory for ketocarotenoids

The present study describes the optimal combination of gene products to deliver astaxanthin. The need to increase the immediate ketocarotenoid precursor β‐carotene in tomato fruit is clearly paramount from the present study and others (Nogueira *et al*., 2017). In the present case a transgene, the lycopene β‐cyclase was introgressed from a line produced previously (D'Ambrosio *et al*., 2004). This approach is complementary to the variant allele of *CYC*‐B used in Nogueira *et al*. (2017). Furthermore, a more effective β‐carotene hydroxylase has been utilized. This combination is similar to that used previously in Huang *et al*. (2013). However, the Huang *et al*. (2013) report negates to demonstrate mendelian inheritance of the chemotype, which has proven to be a vital component of the present study. The initial primary MM:*Crt*ZW transformants produced novel carotenoids in their leaf material with a product profile indicating both CRTW and Z where functional, although the endogenous hydroxylase(s) could also be contributing. Despite the visual evidence in leaf material, confirmed by multi‐platform analytics, no CRTZW derived products above trace levels (total <5 μg/g DW) could be detected in fruit (mature green or ripe). In contrast, the hemizygous progeny produced from segregation of single and double inserts yielded pink mature green fruit in which the endogenous carotenoids were replaced by heterologous ketocarotenoids. To date, the absence of this phenotype in the primary transformants but appearance in subsequent progeny cannot readily be explained. In the homozygous, state loss of vigour leading to unviable plants occurred, presumably as a consequence of high non‐endogenous carotenoid levels interfering with plant processes. Reduced vigour on ketocarotenoid production has been reported previously following transplastomic approaches and subsequent horizontal genome transfer to new species (Lu *et al*., 2017). Industrially, a robust phenotype in a single or double hemizygous state (e.g. from single or double insert parents) is advantageous, readily facilitating hybrid production/vigour. One approach to overcome these detrimental effects in vegetative tissues would be the adoption of tissue specific promoters. However, a contributing factor to the high level ketocarotenoid production would appear to be the initiation of the heterologous pathway through fruit development.

In the majority of cases, metabolic engineering approaches have focused on improving flux through a given pathway. However, the accumulation of newly formed products or overproduction of endogenous metabolites are key aspects for successful prototype development. In the case of tomato, a sink tissue that contains an organelle predisposed to carotenoid accumulation exists. In the present study, the adaption of the plastid was evident from the ultrastructure changes observed. Once again the phenomena of metabolite induced plastid‐differentiation was observed (Fraser *et al*., 2007). In mature “green” fruit producing ketocarotenoids, the ordered chloroplast structure appeared to transition into a “plastoglobuli” containing chromoplast‐like structure. In addition to structural plasticity, the fruit appear to have intrinsic mechanisms to chemically modify non‐endogenous pigments formed. The differential partitioning of ketocarotenoid esters into plastoglobuli resembles mechanisms observed in flowers and *Capsicum* fruits that have adapted to efficient xanthophyll accumulation (Deruere *et al*., 1994). Thus, again the plastid structure is adapting to the metabolites accumulating, a preformed structure into which its cargo is trafficked does not seem feasible.

Non‐food crops are often recommended for the production of speciality chemicals used by industry. However, tomato fruit offers Generally Regarded and Safe Status (GRAS), with improved bioavailability properties and minimal “green” low‐input processing. In contrast non‐food crops typically cannot be utilized as admixes. Instead complex down‐stream processing is required, with unfavourable environmental impact. To date, phenotypic stability in different geographic locations has been demonstrated but scalability remains to be assessed. Based on previous economic evaluations (Nogueira *et al*., 2017), the content of ketocarotenoid in the astaxanthin prototype genotype generated in this study should reach economic competitiveness.

### Why does low level ketocarotenoid production result in lycopene accumulation and delayed ripening?

The dramatic absence of β‐carotene, due to its conversion into ketocarotenoids, was a key feature of the MM:*Crt*ZW tomato lines accumulating lycopene. Potentially, the lycopene accumulation could be a consequence of the absence of feedback inhibition from β‐carotene (or a metabolic product from β‐carotene, Enfissi *et al*., 2017). The expression of transcripts encoding flux controlling biosynthetic enzymes (e.g. *DXS* and *PSY*1, Enfissi *et al*., 2005; Fraser *et al*., 2007) were quantitatively reduced at the onset of ripening. However, a qualitative change in their profile was evident revealing a prolonged expression over ripening. Thus, the duration of carotenoid synthesis appeared extended, providing a plausible explanation for lycopene accumulation. The question then arises why the MM:*Crt*ZW tomato fruit display delayed ripening.

A phytohormone imbalance could have resulted from the perturbations to precursors. For example, reduced β‐carotene levels may reduce abscisic acid levels due to precursor limitations, which in turn prevents the triggering of ethylene to initiate the coordinated features of tomato ripening (Zhang *et al*., 2009). This view is supported by the ability of increased β‐carotene content, concurrently with ketocarotenoid formation, dissipating the delayed over‐ripening (Nogueira *et al*., 2017). An alternative hypothesis for the delay in ripening was revealed from the metabolomics datasets acquired. These data showed a dramatic increase in putrescine and its amino acid derived precursors. Putrescine is a polyamine, these compounds have been shown to act simultaneously with ethylene to control ripening in tomato (Galston and Sawhney, 1990). Previously, targeted engineering to increase polyamines in tomato has generated genotypes with increased lycopene content due to delayed ripening (Mehta *et al*., 2002). However, the delays to ripe fruit are more dramatic in the present study.

The metabolomic signature of the ripe MM:*Crt*ZW fruit with sucrose increased, glucose and fructose reduced, TCA intermediates increased and cell wall derived carbohydrates reduced, also suggest a delay in the biochemical events associated with ripening. However, the increase in sucrose suggests no alteration in the source sink balance has occurred despite reduced photosynthetic capacity. The metabolomics data also revealed a potential explanation for increased tocopherols content in the MM:*Crt*ZW ripe, with chlorophyll reduction correlating with increased phytol (not esterified), the prenyl precursor of tocopherol biosynthesis. These data corroborate the transgenic approached demonstrated previously (Almeida *et al*., 2016).

Recently, it has been proposed that increased fruit antioxidants can act as the progenitor for delayed ripening (Zhang *et al*., 2013). In the MM:*Crt*ZW fruit ascorbic acid, carotenoids, and tocopherols are potent antioxidants representing both hydrophilic and hydrophobic phases that are increased. However, it is unlikely that the proposed mechanisms operate in the present examples because: (i) when β‐carotene is increased by transgenesis or introgression (Nogueira *et al*., 2017) the effects on ripening dissipate and (ii) the *DE‐ETETIOLATED*‐1 down‐regulated plants displaying simultaneous up‐regulation of antioxidants had no effect on ripening (Enfissi *et al*., 2010). Alternatively, it could be the high anthocyanin levels solely that affect the ripening process.

### Lessons learned and biotechnological implications

Since the early 1990s tomato has been subjected to genetic engineering for trait improvement. The first commercial GM (“Flavr Savr”) tomato product occurred in 1994. The last decades have also witnessed rapid advances in the genetic and biochemical resources available in tomato, making it the first choice model for fleshy fruit crops. Concurrently, Systems Biology approaches have captured gene and metabolite regulatory networks/models (Perez‐Fons *et al*., 2014; Foerster *et al*., 2018; Zhu *et al*., 2018). However, despite these technological advances and plausible rationale strategies in place, the fundamental lack of predictability in complex biological systems hinders progress. For example, the changes at a cellular level in response to altered and new metabolites, along with the re‐programming of metabolism experienced in the present study demonstrates the plasticity of the plant cell across multiple levels of cellular regulation, which only become apparent when a perturbation in the system is introduced. Thus, the generic implications from our studies corroborate that biological systems can adapt in a manner not typically observed in engineering disciplines *per se* and, as a consequence, time consuming iterative experimentation remains paramount. The present study has:

1. Identified the combinations of gene products necessary to deliver astaxanthin producing tomato fruit, that exhibit mendelian inheritance of the chemotype.
2. Generated a prototype genotype that overproduces lycopene with extended shelf‐life (or delayed over‐ripening). This material can act as a new commercial source of lycopene formulations.
3. Provided the opportunity to decipher molecular/biochemical mechanisms associated with carotenoid/isoprenoid accumulation and shelf‐life extension concurrently and independently.

## Experimental procedures

### The generation of transgenic tomato plants expressing a β‐carotene ketolase and β‐carotene hydroxylase from *Brevundimonas* sp

The Money Maker variety of tomato was transformed with a construct containing the β‐carotene hydroxylase, *crt*Z, and β‐carotene ketolase, *crt*W, genes from *Brevundimonas* sp., strain SD212 under the control of the 35S promoter (Mortimer *et al*., 2017). A standard *Agrobacterium*‐mediated transformation method with kanamycin selection, as described in Bird *et al*. (1988), was used and positive transformants were confirmed by PCR screening for the transgenes using primers specific for *crt*Z and *crt*W (Table S6). Following acclimation to soil the plants were glasshouse grown (25°C day/15°C night) at Royal Holloway, University of London (Egham, UK), with supplementary lighting (16 h day/8 h night). MM:*Crt*ZW T2 progeny, and HC/MM:*Crt*ZW and HU/MM:*Crt*ZW hybrid lines were also grown in the glasshouse facilities (22–24°C day/15–16°C night) at Agrobios, (Metaponto, Italy), with a light supplementation treatment (three hours in the morning and three in the evening) until the flowering phase.

### Molecular analyses

#### Genotyping

Genomic DNA was extracted from leaf material using Qiagen DNeasy plant mini kit (Qiagen Ltd., Crawley, UK) using the manufacturer's standard protocol. For Southern blotting; genomic DNA (10 μg) was digested using EcoRV and fragments separated on a 0.8% (w/v) agarose gel and then blotted onto positively charged nylon membrane. Blots were probed with a PCR derived NPTII fragment (Table S6) incorporating a digoxigenin‐11‐dUTP label (Roche Diagnostics Ltd., Burgess Hill, UK). A quantitative PCR assay was developed to determine insert number in the T0 or zygosity in subsequent generations based on the ratio of transgene *crt*W to the single locus, homozygous phytoene desaturase (*PDS*) gene. Gene specific primers [designed using Primer3 software (http://primer3.sourceforge.net/) and provided in Table S6] were used to amplify the transgene *crt*W and *PDS* from 25 ng of genomic DNA in a reaction using the QuantiTect SYBR Green real‐time PCR kit (Qiagen, Ltd., Crawley, UK) and a Rotor‐Gene 3000 thermocycler (Qiagen, Ltd.).

#### Gene expression analysis

Total RNA was extracted for use in quantitative real time reverse transcriptase PCR (qRT‐PCR) using Qiagen RNeasy plant mini kit (Qiagen Ltd., Crawley, UK) using the manufacturer's standard protocol including on‐column DNaseI digestion. The QuantiTect SYBR Green, one‐step real‐time RT‐PCR kit (Qiagen Ltd.) was used to determine gene expression levels. Determinations used 25 ng of RNA extracted from a minimum of 3 biological replicates. Reactions were performed on a Rotor‐Gene 3000 thermocycler (Qiagen, Ltd.). Sequencing of PCR products as well as melt curve analysis verified reaction specificity. For quantification, calibration curves were run simultaneously with experimental samples, and Ct calculations were performed by the Rotor‐Gene software using actin as a reference. Primers for quantitative real‐time RT‐PCR were designed using Primer3 software (http://primer3.sourceforge.net/) and are provided in Table S6.

### Biochemical characterization

#### Metabolite analyses

##### Carotenoid and chlorophyll analysis

The extraction of carotenoids and chlorophylls was performed on lyophilized tissue that had been homogenized (Fraser *et al*., 2000). Separation and identification by Thin Layer Chromatography was performed using HPTLC silca gel 60 plates, developed in hexane:ethylacetate 60:40 (v/v) with authentic standards for reference. Separation and detection by high performance liquid chromatography with photodiode array detection (HPLC‐PDA) used a C30 reverse‐phase column (250 × 4.6 mm) purchased from YMC, Wilmington, NC. The solvent system has been described in Fraser *et al*., 2000;. A Waters Alliance model 2695 (Waters Ltd., Watford Herts., UK) injection and solvent delivery system was used with an online PDA (Waters 966, Waters Limited, Watford, Herts, UK). An Acquity ultrahigh performance liquid chromatography system (Waters Ltd.) was used with an Ethylene Bridged Hybrid (BEH C18) column (2.1 × 100 mm, 1.7 μm) and an extended wavelength photodiode array detector (Waters Ltd.) for UPLC‐PDA analysis. The solvent system has been described in detail previously (Nogueira *et al*., 2013). De‐esterification of ketocarotenoid esters was performed using cholesterol esterase from *Pseudomonas* (Sigma, UK) as described in Nogueira *et al*. (2017). Identification was carried out by co‐chromatography and comparison of spectral properties with authentic standards and reference spectra (Fraser *et al*., 2000). Quantitative determination of carotenoids was performed by comparison with dose–response curves (0.2–1.0 μg) constructed from authentic standards.

##### Extraction, derivatization, and GC‐MS analysis of metabolites

Metabolites were routinely extracted from finely milled tomato powder using methanol, with ribitol as an internal standard for relative quantification, and derivatized as described in Enfissi *et al*. (2010). GC‐MS analysis was carried out on an Agilent HP6890 GC with a 5973MSD as described in Enfissi *et al*. (2010) and components identified using a mass spectral (MS) library constructed from in‐house standards as well as the NIST 98 mass spectral library.

#### Subcellular fractionation of plastids

Plastids were isolated from mature green and ripe pericarp tissue and subplastidial fractions separated by discontinuous sucrose density gradient centrifugation as described in Nogueira *et al*. (2013). Fractions (1 mL) were collected, from the top of the gradients using a Minipuls^®^3 peristaltic pump and FC203B fraction collector (Gilson, Dunstable, UK). Individual fractions were analysed for isoprenoid content and verification of subplastidial fractions was performed using antibodies to biomarker proteins.

#### Immunodetection by immunoblot analysis

Proteins were separated by SDS‐PAGE (12.5%) for 3 h at a constant current of 80 mA and electroblotted onto poly(vinylidene difluoride) (PVDF) membranes. Immunodetection was carried out as described in Fraser *et al*. (1994).

#### Transmission electron microscopy of intact plastids

Tomato fruit samples were fixed in 2.5% glutaraldehyde [in 100 mm sodium cacodylate buffer, pH 7.2 (CAB)] and post fixed in 1% osmium tetroxide (in CAB) then sectioned (70 nm) before counterstaining with 4.5% uranyl acetate (in 1% acetic acid) and Reynolds lead citrate as described in Nogueira *et al*. (2013). Sections were viewed in a Jeol 1230 TEM, with an accelerating voltage of 80 kV. Images were recorded with a digital camera. The pictures shown in Figure 3 are representative of 3 biological replicates for each line, from which 12 sections where taken per biological replicate.

#### Determination of *in vivo* chlorophyll fluorescence

*In vivo* chlorophyll fluorescence was determined using a pocket PEA chlorophyll fluorimeter (Hansatech Instruments, King's Lynn, UK). Measurements were recorded with attached leaves. Fluorescence parameters are according to van Kooten and Snel (1990). *F*_v_ */F*_m_ = (*F*_m_ *−F*_0_)/*F*_m_ is the maximum photochemical efficiency of PS II, in the dark‐adapted state.

#### Non‐destructive determination of fruit firmness across development and ripening

A Qualitest™ firmness meter with a 0.25 cm^2^ probe (Qualitest International Inc, USA) was used to accurately determine the firmness factor of fruits, immediately postharvest. The principle of the method is based on the millimetre of probe penetration into the fruit peel, producing a firmness percentage value.

#### Data processing and statistical treatment

All experimentation typically used a minimum of three to six biological and three technical replicates unless stated otherwise. PCA analysis was performed on these data matrixes. SPSS software version 12.01 (SPSS) and SIMCA‐P+ (Umetrics) were used to carry out and display clusters derived from PCA analysis. Student's *t*‐tests were used to determine significant differences between pairwise comparisons among the transgenic lines and their controls. Student's *t*‐tests, means, and standard deviations were all calculated using GraphPad Prism software (GraphPad Software) or Excel (Microsoft) embedded algorithms.

## Conflict of interest

The authors declare no conflict of interest.

## Data availability

All relevant data are available within the manuscript.

## Author contributions

All authors contributed to the conception of the project. PDF, EMAE, and GG obtained funding; NM provided vital materials; EMAE, MN, CDA, ALS, GG, and PDF carried out the experimental work. All authors contributed to the writing and approval of the manuscript.

## Supporting information


**Figure S1** Thin layer chromatographic separation of pigments from MM and MM:*Crt*ZW leaf tissue.
**Figure S2** Principal Component Analysis of leaf, mature green and ripe fruit tissues from MM:*Crt*ZW plants relative to the azygous control.
**Figure S3** HPLC chromatograms at 470 nm of ripe fruit extracts from (a) HC/MM:*Crt*ZW and (b) HU/MM:*Crt*ZW.
**Figure S4** Phenotypes of HC/MM:*Crt*ZW and HU/MM:*Crt*ZW compared to MM:*Crt*ZW.
**Table S1a** Levels of endogenous leaf pigments in T0 plants transformed with *Brevundimonas* sp. *crt*Z and *crt*W.
**Table S1b** Levels of ketocarotenoids in leaf tissue from T0 plants transformed with *crt*Z and *crt*W *Brevundimonas* sp.
**Table S2** Retention times and spectral characteristics (in the eluting solvent) used in identification of isoprenoids separated by HPLC‐PDA and TLC.
**Table S3a** Levels of endogenous leaf pigments in T1 plants transformed with *Brevundimonas* sp. *crt*Z and *crt*W.
**Table S3b** Levels of novel ketocarotenoids in leaf tissue from T1 plants transformed with *Brevundimonas* sp. *crt*Z and *crt*W.
**Table S3c** Levels of endogenous mature green fruit pigments in T1 plants transformed with *Brevundimonas* sp. *crt*Z and *crt*W.
**Table S3d** Levels of novel ketocarotenoids in mature green fruit from T1 plants transformed with *Brevundimonas* sp. *crt*Z and *crt*W.
**Table S3e** Levels of endogenous ripe fruit pigments in T1 plants transformed with *Brevundimonas* sp. *crt*Z and *crt*W.
**Table S3f** Levels of novel ketocarotenoids in ripe fruit from T1 plants transformed with *Brevundimonas* sp. *crt*Z and *crt*W.
**Table S4a** Levels of endogenous leaf pigments in T2 plants transformed with *Brevundimonas* sp. *crt*Z and *crt*W.
**Table S4b** Levels of novel ketocarotenoids in leaf tissue from T2 plants transformed with *Brevundimonas* sp. *crt*Z and *crt*W.
**Table S4c** Pigment levels across 5 stages of fruit development and ripening in T2 plants transformed with *Brevundimonas* sp. *crt*Z and *crt*W.
**Table S5** Determination of fruit softening across ripening in T2 plants transformed with Brevundimonas sp. *crt*Z and *crt*W.
**Table S6** Sequences of primers used in real‐time RT‐PCR and PCR.
