# Supplementary material for: The road to astaxanthin production in tomato fruit reveals plastid and metabolic adaptation resulting in an unintended high lycopene genotype with delayed over‐ripening properties
Source: Plant Biotechnol J. 2019 Feb 1;17(8):1501–13. doi: 10.1111/pbi.13073 (PMC6662112; doi:10.1111/pbi.13073)
Supplement: Supplementary file 1 — Figure S1 Thin layer chromatographic separation of pigments from MM and MM:CrtZW leaf tissue. Figure S2 Principal Component Analysis of leaf, mature green and ripe fruit tissues from MM:CrtZW plants relative to the azygous control. Figure S3 HPLC chromatograms at 470 nm of ripe fruit extracts from (a) HC/MM:CrtZW and (b) HU/MM:CrtZW. Figure S4 Phenotypes of HC/MM:CrtZW and HU/MM:CrtZW compared to MM:CrtZW. Table S1a Levels of endogenous leaf pigments in T0 plants transformed with Brevundimonas sp. crtZ and crtW. Table S1b Levels of ketocarotenoids in leaf tissue from T0 plants transformed with crtZ and crtW Brevundimonas sp. Table S2 Retention times and spectral characteristics (in the eluting solvent) used in identification of isoprenoids separated by HPLC‐PDA and TLC. Table S3a Levels of endogenous leaf pigments in T1 plants transformed with Brevundimonas sp. crtZ and crtW. Table S3b Levels of novel ketocarotenoids in leaf tissue from T1 plants transformed with Brevundimonas sp. crtZ and crtW. Table S3c Levels of endogenous mature green fruit pigments in T1 plants transformed with Brevundimonas sp. crtZ and crtW. Table S3d Levels of novel ketocarotenoids in mature green fruit from T1 plants transformed with Brevundimonas sp. crtZ and crtW. Table S3e Levels of endogenous ripe fruit pigments in T1 plants transformed with Brevundimonas sp. crtZ and crtW. Table S3f Levels of novel ketocarotenoids in ripe fruit from T1 plants transformed with Brevundimonas sp. crtZ and crtW. Table S4a Levels of endogenous leaf pigments in T2 plants transformed with Brevundimonas sp. crtZ and crtW. Table S4b Levels of novel ketocarotenoids in leaf tissue from T2 plants transformed with Brevundimonas sp. crtZ and crtW. Table S4c Pigment levels across 5 stages of fruit development and ripening in T2 plants transformed with Brevundimonas sp. crtZ and crtW. Table S5 Determination of fruit softening across ripening in T2 plants transformed with Brevundimonas sp. crtZ and crtW. Table S6 Seque [file PBI-17-1501-s001.docx]

**Supplementary Table 1a.** Levels of endogenous leaf pigments in T0 plants transformed with *Brevundimonas* sp. *crt*Z and *crt*W.

| Line | β-Carotene  (μg/g DW) | Lutein  (μg/g DW) | Violaxanthin (μg/g DW) | Neoxanthin (μg/g DW) | Chlorophyll a (mg/g DW) | Chlorophyll b (mg/g DW) |
| --- | --- | --- | --- | --- | --- | --- |
| MM | 802.5 ± 42.9 | 1226.3 ± 60.8 | 319.7 ± 32.5 | 297.4 ± 11.1 | 27.7 ± 1.1 | 8.0 ± 0.9 |
| MM:*Crt*ZW1 | **193.2 ± 15.8***** | **132.0 ± 12.2***** | n/d | n/d | **18.7 ± 1.0***** | **5.7 ± 0.4*** |
| MM:*Crt*ZW2 | **211.2 ± 12.9***** | **157.6 ± 9.9***** | n/d | n/d | **19.3 ± 1.9**** | 6.2 ± 0.5 |
| MM:*Crt*ZW3 | **302.7 ± 13.5***** | **252.3 ± 28.3***** | n/d | n/d | **22.6 ± 2.0*** | 7.4 ± 0.2 |
| MM:*Crt*ZW4 | **283.6 ± 23.5***** | **233.4 ± 23.1***** | n/d | n/d | **21.2 ± 2.0**** | 7.3 ± 0.3 |
| MM:*Crt*ZW5 | **265.3 ± 19.2***** | **207.5 ± 21.5***** | n/d | n/d | **20.7 ± 1.9**** | 6.5 ± 0.2 |
| MM:*Crt*ZW6 | 725.9 ± 15.1 | 1172.8 ± 16.4 | 234.5 ± 14.4 | 213.2 ± 10.5 | 26.5 ± 1.3 | 7.9 ± 0.4 |
| MM:*Crt*ZW7 | **281.1 ± 18.3***** | **227.3 ± 30.4***** | n/d | n/d | **22.2 ± 2.3*** | **6.11± 0.4*** |
| MM:*Crt*ZW8 | **318.9 ± 27.5***** | **276.4 ± 26.3***** | n/d | n/d | 23.6 ± 2.1 | 7.0 ± 0.6 |
| MM:*Crt*ZW9 | **254.0 ± 17.7***** | **198.7 ± 19.3***** | n/d | n/d | **20.5 ± 1.7**** | 6.8 ± 0.5 |
| MM:*Crt*ZW10 | **359.4 ± 19.3***** | **294.8 ± 24.3***** | n/d | n/d | **23.1 ± 1.4**** | 7.2 ± 0.4 |
| MM:*Crt*ZW11 | **239.0 ± 13.5***** | **181.6 ± 16.8***** | n/d | n/d | **19.7 ± 1.4***** | 6.8 ± 0.41 |
| MM:*Crt*ZW12 | **321.6 ± 10.2***** | **279.8 ± 12.8***** | n/d | n/d | **23.0 ± 1.8*** | 7.6 ± 0.5 |

The values shown are the mean ± standard deviation, where n=3 to 10. Significant differences, shown in bold, between the mean of MM:*Crt*ZW and the MM control, for each compound were evaluated using the Student’s *t*-tests (*, P< 0.05 ; **, P< 0.01 ; ***, P<0.001). n/d: Not detected.

**Supplementary Table 1b.** Levels of ketocarotenoids in leaf tissue from T0 plants transformed with *crt*Z and *crt*W *Brevundimonas* sp.

| Line | Echinonone  (µg/g DW) | 3’-OH-Echinonone (µg/g DW) | Canthaxanthin (µg/g DW) | Phoenicoxanthin (µg/g DW) | Astaxanthin (µg/g DW) |
| --- | --- | --- | --- | --- | --- |
| MM | n/d | n/d | n/d | n/d | n/d |
| MM:*Crt*ZW1 | 20.31 ± 1.21 | 28.17 ± 1.65 | 385.65 ± 20.45 | 3.79 ± 0.08 | 11.94 ± 0.96 |
| MM:*Crt*ZW2 | 19.61 ± 0.98 | 32.26 ± 1.44 | 392.12 ± 18.47 | 3.82 ± 0.09 | 11.75 ± 0.94 |
| MM:*Crt*ZW3 | 22.18 ± 1.11 | 38.88 ± 1.38 | 375.41 ± 21.61 | 4.45 ± 0.04 | 12.30 ± 0.83 |
| MM:*Crt*ZW4 | 22.65 ± 1.15 | 36.51 ± 1.77 | 374.06 ± 10.93 | 4.24 ± 0.05 | 12.77 ± 0.77 |
| MM:*Crt*ZW5 | 19.40 ± 1.01 | 35.03 ± 0.89 | 381.18 ± 11.23 | 4.60 ± 0.05 | 12.48 ± 0.85 |
| MM:*Crt*ZW6 | n/d | n/d | n/d | n/d | n/d |
| MM:*Crt*ZW7 | 20.87 ± 1.20 | 35.56 ± 1.47 | 340.98 ± 22.58 | 3.97 ± 0.11 | 11.62 ± 0.62 |
| MM:*Crt*ZW8 | 23.99 ± 0.96 | 40.23 ± 1.31 | 386.76 ± 20.59 | 4.03 ± 0.19 | 11.73 ± 0.69 |
| MM:*Crt*ZW9 | 18.06 ± 1.33 | 29.79 ± 2.01 | 332.81 ± 21.17 | 4.81 ± 0.12 | 12.06 ± 0.71 |
| MM:*Crt*ZW10 | 17.63 ± 1.17 | 31.00 ± 1.22 | 352.48 ± 18.18 | 3.89 ± 0.08 | 11.71 ± 0.55 |
| MM:*Crt*ZW11 | 18.54 ± 0.65 | 29.36 ± 1.24 | 366.92 ± 16.39 | 4.11 ± 0.09 | 11.93 ± 0.57 |
| MM:*Crt*ZW12 | 23.23 ± 0.45 | 38.05 ± 1.19 | 394.50 ± 17.08 | 4.96 ± 0.06 | 12.37 ± 0.99 |

The values shown are the mean ± standard deviation, where n=3 to 10. n/d: Not detected.

| Isoprenoid | Retention time (min) HPLC | Spectral characteristics (nm at λ_max_) | Retention time (min) UPLC | Spectral characteristics (nm at λ_max_) | TLC system I  R_F_ | TLC system II  R_F_ | TLC system III R_F_ |
| --- | --- | --- | --- | --- | --- | --- | --- |
| 15-*cis*-phytoene | 21.93 | 275.8, 286.4, 297.3 | 6.28 | 286.2 | 0.6 to 0.8 | 0.7 | NR |
| *trans-*Phytofluene | 23.08 | 330.1, 348.2, 365.8 | NR | NR | 0.6 to 0.8 | 0.6 | NR |
| *cis-*Phytofluene | 24.48 | 330.1, 348.2, 365.8 | 8.16 | -, 349.0, 368.3 | 0.6 to 0.8 | 0.6 | NR |
| *cis*-ζ-Carotene | 27.55 | 380.1, 401.8, 427.0 | 7.89 | -, 400.8, 425.0 | 0.6 to 0.8 | 0.3 | NR |
| All-*trans*-Lycopene | 41.78 | 446.3, 472.9, 504.4 | 7.33 | -, 472.3, 502.7 | 0.4 | NR | NR |
| β-Carotene | 26.92 | -, 452.4, 480.2 | 8.07 | -, 454.1, 479.6 | 0.5 to 0.8 | 0.5 | 0.77 |
| Lutein | 14.50 | 421.0, 445.1, 472.9 | 4.33 | -, 448.0, 476.0 | NR | NR | NR |
| Violaxanthin | 10.38 | 417.4, 442.1, 471.7 | 2.46 | 417.7, 442.0, 471.1 | NR | NR | NR |
| Neoxanthin | 10.94 | 416.2, 440.3, 470.5 | 2.32 | 414.1, 438.3, 467.4 | NR | NR | NR |
| Chlorophyll a | 17.75 | 433.1, 657.0 | 7.23 | 429.8 | NR | NR | NR |
| Chlorophyll b | 13.47 | 466.9, 656.0 | 6.90 | 456.5 | NR | NR | NR |
| Pheophytin A | 26.75 | 409 | 8.05 | 409.3 | NR | NR | NR |
| Echinenone | 22.51 | 464.5 | 6.97 | 460.2 | NR | NR | 0.65 |
| 3-Hydroxyechinenone | 22.00 | 464.5 | 6.77 | 465 | NR | NR | 0.51 |
| Canthaxanthin | 17.49 | 476.6 | 5.86 | 477.2 | NR | NR | 0.35 |
| Phoenicoxanthin | 16.13 | 475.3 | 4.57 | 479.6 | NR | NR | 0.23 |
| Adonixanthin | 15.61 | 463.2 | 3.68 | 466.2 | NR | NR | NR |
| 4-Ketoantheraxanthin | 12.7 | 454.8,470.5 | 2.63 | 455.3, 473.5 | NR | NR | NR |
| Astaxanthin | 15.36 | 476.6 | 3.15 | 479.6 | NR | NR | 0.20 |

**Supplementary Table 2.** Retention times and spectral characteristics (in the eluting solvent) used in identification of isoprenoids separated by HPLC-PDA and TLC.

TLC system I comprised of 15% Toluene in Petroleum-ether 40-60^o^C, TLC system II comprised of 3% Toluene in Petroleum-ether 40-60^o^C, TLC system III comprised of Hexane:Ethylacetate 60:40 (v/v). NR: not recorded; NA: not applicable.

**Supplementary Table 3a.** Levels of endogenous leaf pigments in T1 plants transformed with *Brevundimonas* sp. *crt*Z and *crt*W.

| Line | Zyg | β-Carotene  (µg/g DW) | | | Lutein  (µg/g DW) | | | Violaxanthin  (µg/g DW) | | | Neoxanthin  (µg/g DW) | | | Chlorophyll a (mg/g DW) | | | Chlorophyll b (mg/g DW) | | |
| --- | --- | --- | --- | --- | --- | --- | --- | --- | --- | --- | --- | --- | --- | --- | --- | --- | --- | --- | --- |
| MM | WT | 818.0 | ± | 49.9 | 1292.0 | ± | 392.4 | 328.8 | ± | 22.7 | 308.4 | ± | 14.9 | 29.4 | ± | 0.7 | 9.4 | ± | 0.3 |
| MM:*Crt*ZW10-1 | Hemi | **276.6** | ± | **42.1***** | **221.6** | ± | **76.4*** | n/d | | | n/d | | | **25.1** | ± | **1.2**** | 9.2 | ± | 0.3 |
| MM:*Crt*ZW10-4 | Hemi | **263.3** | ± | **2.9**** | **180.6** | ± | **23.0*** | n/d | | | n/d | | | **22.2** | ± | **0.5***** | **7.7** | ± | **0.2**** |
| MM:*Crt*ZW10-5 | Azy | **647.5** | ± | **35.1**** | 1520.8 | ± | 93.6 | 354.0 | ± | 21.7 | 330.2 | ± | 20.6 | **25.6** | ± | **1.4*** | 8.8 | ± | 0.4 |
| MM:*Crt*ZW10-6 | Azy | **631.0** | ± | **17.3**** | 1518.4 | ± | 25.9 | 312.2 | ± | 4.5 | 311.7 | ± | 8.6 | **25.3** | ± | **1.1**** | 9.0 | ± | 0.3 |
| MM:*Crt*ZW10-9 | Azy | **575.3** | ± | **41.0**** | 1365.3 | ± | 89.8 | 347.2 | ± | 31.8 | 312.8 | ± | 34.8 | **24.7** | ± | **1.8*** | **8.3** | ± | **0.3**** |
| MM:*Crt*ZW10-11 | Hemi | **242.2** | ± | **6.9**** | **184.4** | ± | **42.3*** | n/d | | | n/d | | | **20.1** | ± | **3.3*** | 6.8 | ± | 1.3 |
| MM:*Crt*ZW10-14 | Hemi | **260.8** | ± | **21.4***** | **207.7** | ± | **23.9*** | n/d | | | n/d | | | **23.7** | ± | **0.7***** | **8.6** | ± | **0.3*** |
| MM:*Crt*ZW10-16 | Hemi | **288.7** | ± | **11.2**** | **203.2** | ± | **11.4*** | n/d | | | n/d | | | **23.5** | ± | **0.2**** | **7.8** | ± | **0.1**** |
| MM:*Crt*ZW10-17 | Hemi | **256.7** | ± | **3.4**** | **205.6** | ± | **20.1*** | n/d | | | n/d | | | **24.6** | ± | **1.3**** | 9.0 | ± | 0.5 |
| MM:*Crt*ZW12-4 | Azy | 752.2 | ± | 85.0 | 1882.9 | ± | 66.2 | 362.7 | ± | 10.9 | 330.2 | ± | 9.4 | 30.7 | ± | 2.4 | 11.9 | ± | 1.2 |
| MM:*Crt*ZW12-5 | Hemi | **231.9** | ± | **9.9**** | **189.4** | ± | **16.3*** | n/d | | | n/d | | | **22.0** | ± | **2.8*** | 8.2 | ± | 1.2 |
| MM:*Crt*ZW12-7 | Azy | **656.7** | ± | **18.6*** | 1677.2 | ± | 170.9 | 332.9 | ± | 18.0 | 334.2 | ± | 20.2 | **24.7** | ± | **1.4**** | 9.3 | ± | 0.2 |
| MM:*Crt*ZW12-8 | Hemi | **260.0** | ± | **8.1**** | **217.4** | ± | **10.9*** | n/d | | | n/d | | | **24.6** | ± | **0.5***** | **8.8** | ± | **0.1*** |
| MM:*Crt*ZW12-9 | Azy | **661.5** | ± | **33.8**** | 1695.9 | ± | 219.0 | 350.1 | ± | 36.6 | 324.6 | ± | 37.2 | 27.8 | ± | 1.1 | **10.3** | ± | **0.1*** |

The values shown are the mean ± standard deviation, where n=3 to 10. Significant differences, shown in bold, between the mean of MM:*Crt*ZW and the MM control, for each compound were evaluated using the Student’s *t*-tests (*, P< 0.05 ; **, P< 0.01 ; ***, P<0.001). n/d: Not detected; Zyg: zygosity; WT: wild type; Azy: azygous; Hemi: hemizygous; Hom: homozygous.

**Supplementary Table 3b.** Levels of novel ketocarotenoids in leaf tissue from T1 plants transformed with *Brevundimonas* sp. *crt*Z and *crt*W.

| Line | Zyg | 3’-OH Echinenone  (µg/g DW) | | | Canthaxanthin (µg/g DW) | | | Phoenicoxanthin (µg/g DW) | | | Astaxanthin  (µg/g DW) | | | Phoenicoxanthin Ester (µg/g DW) | | |
| --- | --- | --- | --- | --- | --- | --- | --- | --- | --- | --- | --- | --- | --- | --- | --- | --- |
| MM | WT | n/d | | | n/d | | | n/d | | | n/d | | | n/d | | |
| MM:*Crt*ZW10-1 | Hemi | 64.2 | ± | 7.9 | 647.9 | ± | 11.1 | 6.6 | ± | 0.3 | 16.8 | ± | 2.7 | 1.1 | ± | 0.04 |
| MM:*Crt*ZW10-4 | Hemi | 42.9 | ± | 3.2 | 463.7 | ± | 23.9 | 5.6 | ± | 0.3 | 17.5 | ± | 0.7 | 1.5 | ± | 0.1 |
| MM:*Crt*ZW10-5 | Azy | n/d | | | n/d | | | n/d | | | n/d | | | n/d | | |
| MM:*Crt*ZW10-6 | Azy | n/d | | | n/d | | | n/d | | | n/d | | | n/d | | |
| MM:*Crt*ZW10-9 | Azy | n/d | | | n/d | | | n/d | | | n/d | | | n/d | | |
| MM:*Crt*ZW10-11 | Hemi | 42.2 | ± | 8.2 | 407.8 | ± | 73.7 | 5.6 | ± | 0.9 | 13.3 | ± | 3.1 | 1.2 | ± | 0.4 |
| MM:*Crt*ZW10-14 | Hemi | 45.7 | ± | 5.3 | 484.0 | ± | 27.7 | 6.1 | ± | 0.1 | 15.6 | ± | 4.8 | 1.0 | ± | 0.1 |
| MM:*Crt*ZW10-16 | Hemi | 47.0 | ± | 2.2 | 419.8 | ± | 32.9 | 6.6 | ± | 0.1 | 16.2 | ± | 2.2 | 1.2 | ± | 0.1 |
| MM:*Crt*ZW10-17 | Hemi | 52.9 | ± | 1.9 | 569.4 | ± | 40.9 | 7.0 | ± | 0.4 | 18.9 | ± | 1.6 | 1.1 | ± | 0.04 |
| MM:*Crt*ZW12-4 | Azy | n/d | | | n/d | | | n/d | | | n/d | | | n/d | | |
| MM:*Crt*ZW12-5 | Hemi | 54.1 | ± | 8.4 | 565.0 | ± | 87.5 | 6.5 | ± | 1.0 | 15.3 | ± | 1.1 | 1.0 | ± | 0.2 |
| MM:*Crt*ZW12-7 | Azy | n/d | | | n/d | | | n/d | | | n/d | | | n/d | | |
| MM:*Crt*ZW12-8 | Hemi | 60.6 | ± | 2.4 | 611.6 | ± | 21.8 | 7.3 | ± | 0.4 | 15.6 | ± | 1.1 | 1.3 | ± | 0.1 |
| MM:*Crt*ZW12-9 | Azy | n/d | | | n/d | | | n/d | | | n/d | | | n/d | | |

The values shown are the mean ± standard deviation, where n=3 to 10. n/d: Not detected; Zyg: zygosity; WT: wild type; Azy: azygous; Hemi: hemizygous; Hom: homozygous.

**Supplementary Table 3c.** Levels of endogenous mature green fruit pigments in T1 plants transformed with *Brevundimonas* sp. *crt*Z and *crt*W.

| Line | Zyg | β-carotene  (µg/g DW) | | | Lutein  (µg/g DW) | | | Chlorophyll a  (mg/g DW) | | | Chlorophyll b  (µg/g DW) | | |
| --- | --- | --- | --- | --- | --- | --- | --- | --- | --- | --- | --- | --- | --- |
| MM | WT | 4.3 | ± | 0.5 | 21.4 | ± | 4.3 | 3.9 | ± | 6.7 | 167.5 | ± | 26.0 |
| MM:*Crt*ZW10-1 | Hemi | n/d | | | n/d | | | **1.8** | ± | **0.2**** | **95.7** | ± | **9.9**** |
| MM:*Crt*ZW10-4 | Azy | 6.2 | ± | 0.7 | **33.4** | ± | **3.2*** | 5.7 | ± | 0.4 | **229.6** | ± | **14.8**** |
| MM:*Crt*ZW10-5 | Hom | 3.0 | ± | 0.4 | 12.6 | ± | 2.9 | 2.5 | ± | 0.2 | **119.7** | ± | **10.0*** |
| MM:*Crt*ZW10-6 | Azy | 4.5 | ± | 0.2 | 20.9 | ± | 2.8 | 3.7 | ± | 0.01 | 155.3 | ± | 2.1 |
| MM:*Crt*ZW10-9 | Azy | 3.5 | ± | 0.6 | 22.3 | ± | 0.02 | 3.3 | ± | 0.2 | 164.7 | ± | 1.9 |
| MM:*Crt*ZW10-10 | Hom | 3.3 | ± | 0.3 | **12.0** | ± | **2.2*** | 3.1 | ± | 0.06 | 136.0 | ± | 0.7 |
| MM:*Crt*ZW10-14 | Azy | 7.7 | ± | 0.9 | 32.7 | ± | 5.7 | **6.2** | ± | **0.2**** | **279.7** | ± | **2.2**** |
| MM:*Crt*ZW10-16 | Hemi | n/d | | | n/d | | | **0.8** | ± | **0.03**** | **85.5** | ± | **1.85**** |
| MM:*Crt*ZW10-17 | Hemi | n/d | | | n/d | | | **1.1** | ± | **0.04**** | **102.0** | ± | **6.2**** |
| MM:*Crt*ZW12-4 | Hemi | n/d | | | n/d | | | **1.2** | ± | **0.05**** | **71.2** | ± | **5.6**** |
| MM:*Crt*ZW12-5 | Azy | **7.2** | ± | **0.1***** | 33.6 | ± | 10.4 | **6.4** | ± | **0.2**** | **265.3** | ± | **3.7**** |
| MM:*Crt*ZW12-7 | Hemi | n/d | | | n/d | | | **1.4** | ± | **0.05**** | **86.8** | ± | **6.0**** |
| MM:*Crt*ZW12-8 | Azy | 3.9 | ± | 0.7 | 22.3 | ± | 0.5 | 3.0 | ± | 0.01 | 160.9 | ± | 6.2 |
| MM:*Crt*ZW12-9 | Hemi | n/d | | | n/d | | | **1.5** | ± | **0.02**** | **97.2** | ± | **0.6**** |
| MM:*Crt*ZW12-10 | Azy | 5.8 | ± | 0.5 | 25.6 | ± | 2.7 | 4.7 | ± | 0.4 | 191.4 | ± | 14.2 |
| MM:*Crt*ZW12-11 | Hemi | n/d | | | n/d | | | **1.1** | ± | **0.2***** | **56.8** | ± | **10.6***** |
| MM:*Crt*ZW12-12 | Hemi | n/d | | | n/d | | | **1.3** | ± | **0.04**** | **100.0** | ± | **20.8*** |
| MM:*Crt*ZW12-14 | Azy | **2.7** | ± | **0.1**** | **14.1** | ± | **2.1*** | **2.2** | ± | **0.03**** | **108.2** | ± | **0.5*** |

The values shown are the mean ± standard deviation, where n=3 to 10. Significant differences, shown in bold, between the mean of MM:*Crt*ZW and the MM control, for each compound were evaluated using the Student’s *t*-tests (*, P< 0.05 ; **, P< 0.01 ; ***, P<0.001). n/d: Not detected; Zyg: zygosity; WT: wild type; Azy: azygous; Hemi: hemizygous; Hom: homozygous.

**Supplementary Table 3d.** Levels of novel ketocarotenoids in mature green fruit from T1 plants transformed with *Brevundimonas* sp. *crt*Z and *crt*W.

| Line | Zyg | Echinenone (µg/g DW) | | | Canthaxanthin  (µg/g DW) | | | Phoenicoxanthin (µg/g DW) | | | Astaxanthin (µg/g DW) | | | Phoenicoxanthin Ester (µg/g DW) | | |
| --- | --- | --- | --- | --- | --- | --- | --- | --- | --- | --- | --- | --- | --- | --- | --- | --- |
| MM | WT | n/d | | | n/d | | | n/d | | | n/d | | | n/d | | |
| MM:*Crt*ZW10-1 | Hemi | 0.7 | ± | 0.1 | 2.6 | ± | 0.4 | 0.4 | ± | 0.1 | 5.3 | ± | 3.0 | 0.06 | ± | 0.001 |
| MM:*Crt*ZW10-4 | Azy | n/d | | | n/d | | | n/d | | | n/d | | | n/d | | |
| MM:*Crt*ZW10-5 | Hom | n/d | | | n/d | | | n/d | | | n/d | | | n/d | | |
| MM:*Crt*ZW10-6 | Azy | n/d | | | n/d | | | n/d | | | n/d | | | n/d | | |
| MM:*Crt*ZW10-9 | Azy | n/d | | | n/d | | | n/d | | | n/d | | | n/d | | |
| MM:*Crt*ZW10-10 | Hom | n/d | | | n/d | | | n/d | | | n/d | | | n/d | | |
| MM:*Crt*ZW10-14 | Azy | n/d | | | n/d | | | n/d | | | n/d | | | n/d | | |
| MM:*Crt*ZW10-16 | Hemi | 0.7 | ± | 0.1 | 2.5 | ± | 0.1 | 0.5 | ± | 0.04 | 9.8 | ± | 0.3 | 0.34 | ± | 0.02 |
| MM:*Crt*ZW10-17 | Hemi | 0.7 | ± | 0.04 | 3.2 | ± | 0.1 | 0.4 | ± | 0.1 | 7.7 | ± | 0.8 | 0.37 | ± | 0.01 |
| MM:*Crt*ZW12-4 | Hemi | 0.8 | ± | 0.1 | 3.9 | ± | 0.2 | 0.6 | ± | 0.04 | 9.1 | ± | 0.3 | 0.34 | ± | 0.03 |
| MM:*Crt*ZW12-5 | Azy | n/d | | | n/d | | | n/d | | | n/d | | | n/d | | |
| MM:*Crt*ZW12-7 | Hemi | 0.8 | ± | 0.2 | 3.2 | ± | 0.7 | 0.6 | ± | 0.1 | 9.5 | ± | 0.9 | 0.25 | ± | 0.03 |
| MM:*Crt*ZW12-8 | Azy | n/d | | | n/d | | | n/d | | | n/d | | | n/d | | |
| MM:*Crt*ZW12-9 | Hemi | 1.0 | ± | 0.1 | 4.1 | ± | 0.2 | 0.6 | ± | 0.04 | 9.1 | ± | 0.5 | 0.22 | ± | 0.14 |
| MM:*Crt*ZW12-10 | Azy | n/d | | | n/d | | | n/d | | | n/d | | | n/d | | |
| MM:*Crt*ZW12-11 | Hemi | 0.4 | ± | 0.02 | 1.4 | ± | 0.1 | 0.2 | ± | 0.04 | 1.6 | ± | 0.001 | 0.22 | ± | 0.01 |
| MM:*Crt*ZW12-12 | Hemi | 0.8 | ± | 0.1 | 3.5 | ± | 0.1 | 0.6 | ± | 0.1 | 9.0 | ± | 0.5 | 0.20 | ± | 0.05 |
| MM:*Crt*ZW12-14 | Azy | n/d | | | n/d | | | n/d | | | n/d | | | n/d | | |

The values shown are the mean ± standard deviation, where n=3 to 10. n/d: Not detected; Zyg: zygosity; WT: wild type; Azy: azygous; Hemi: hemizygous; Hom: homozygous.

**Supplementary Table 3e.** Levels of endogenous ripe fruit pigments in T1 plants transformed with *Brevundimonas* sp. *crt*Z and *crt*W.

| Line | Zyg | Phytoene  (µg/g DW) | | | Phytofluene  (µg/g DW) | | | Lycopene  (µg/g DW) | | | β-Carotene  (µg/g DW) | | | Lutein  (µg/g DW) | | |
| --- | --- | --- | --- | --- | --- | --- | --- | --- | --- | --- | --- | --- | --- | --- | --- | --- |
| MM | WT | 108.8 | ± | 17.8 | 232.2 | ± | 17.0 | 763.1 | ± | 25.7 | 228.37 | ± | 21.8 | 24.8 | ± | 6.0 |
| MM:*Crt*ZW10-1 | Hemi | 118.3 | ± | 0.9 | 232.5 | ± | 20.4 | 562.6 | ± | 58.6 | **69.8** | ± | **2.3**** | n/d | | |
| MM:*Crt*ZW10-4 | Azy | 653.1 | ± | 84.1 | 510.6 | ± | 48.9 | 747.6 | ± | 51.5 | 219.2 | ± | 9.9 | 13.6 | ± | 3.7 |
| MM:*Crt*ZW10-5 | Hom | **67.6** | ± | **2.3*** | 178.3 | ± | 20.0 | **216.9** | ± | **36.1**** | 169.3 | ± | 23.9 | **2.7** | ± | **0.7*** |
| MM:*Crt*ZW10-6 | Azy | **226.4** | ± | **20.4*** | **376.1** | ± | **20.5*** | 1379.3 | ± | 181.6 | **251.3** | ± | **4.6*** | 19.6 | ± | 3.6 |
| MM:*Crt*ZW10-9 | Azy | **173.3** | ± | **12.4**** | 262.53 | ± | 24.3 | 1078.1 | ± | 73.2 | 251.8 | ± | 24.0 | 17.3 | ± | 4.8 |
| MM:*Crt*ZW10-10 | Hom | **215.0** | ± | **9.7***** | 306.1 | ± | 35.2 | **1972.8** | ± | **72.5**** | 222.8 | ± | 35.6 | 14.9 | ± | 3.3 |
| MM:*Crt*ZW10-14 | Azy | **565.7** | ± | **2.1***** | **462.3** | ± | **5.6***** | 934.4 | ± | 20.6 | 208.0 | ± | 9.6 | **6.9** | ± | **1.9*** |
| MM:*Crt*ZW10-16 | Hemi | **233.7** | ± | **16.7**** | 337.7 | ± | 36.6 | **1260.1** | ± | **8.9**** | **49.8** | ± | **3.3**** | n/d | | |
| MM:*Crt*ZW10-17 | Hemi | **217.7** | ± | **15.0**** | **364.9** | ± | **25.7**** | **2832.0** | ± | **56.4***** | **37.0** | ± | **4.1**** | n/d | | |
| MM:*Crt*ZW12-4 | Hemi | **225.2** | ± | **17.2**** | **339.3** | ± | **14.7**** | **2518.3** | ± | **19.7***** | **62.6** | ± | **3.8**** | n/d | | |
| MM:*Crt*ZW12-5 | Azy | **64.0** | ± | **6.2**** | **205.9** | ± | **5.2*** | **246.4** | ± | **58.9*** | **201.5** | ± | **2.8*** | **4.1** | ± | **3.7*** |
| MM:*Crt*ZW12-7 | Hemi | **262.2** | ± | **2.49***** | **342.2** | ± | **11.9**** | **1344.8** | ± | **23.1**** | **36.8** | ± | **0.2***** | n/d |  |  |
| MM:*Crt*ZW12-8 | Azy | 165.6 | ± | 33.6 | 262.5 | ± | 10.6 | 537.8 | ± | 154.5 | 228.2 | ± | 4.4 | **5.0** | ± | **0.3** |
| MM:*Crt*ZW12-9 | Hemi | 132.6 | ± | 14.1 | 256.5 | ± | 34.6 | 502.1 | ± | 80.3 | **39.6** | ± | **2.8**** | n/d |  |  |
| MM:*Crt*ZW12-10 | Azy | 83.5 | ± | 12.3 | 183.4 | ± | 29.0 | 440.3 | ± | 218.6 | 179.7 | ± | 33.4 | **7.0** | ± | **0.9*** |
| MM:*Crt*ZW12-11 | Hemi | **279.1** | ± | **13.5***** | **332.1** | ± | **7.8***** | 1475.1 | ± | 163.8 | **47.2** | ± | **1.5**** | n/d | | |
| MM:*Crt*ZW12-12 | Hemi | **256.1** | ± | **15.5**** | **380.9** | ± | **20.3***** | **2668.4** | ± | **60.0***** | **54.0** | ± | **2.1**** | n/d | | |
| MM:*Crt*ZW12-14 | Azy | 204.7 | ± | 26.5 | 260.7 | ± | 11.0 | 589.1 | ± | 104.2 | **205.4** | ± | **4.4*** | **4.1** | ± | **0.1*** |

The values shown are the mean ± standard deviation, where n=3 to 10. Significant differences, shown in bold, between the mean of MM:*Crt*ZW and the MM control, for each compound were evaluated using the Student’s *t*-tests (*, P< 0.05 ; **, P< 0.01 ; ***, P<0.001). n/d: Not detected; Zyg: zygosity; WT: wild type; Azy: azygous; Hemi: hemizygous; Hom: homozygous.

| Line | Zyg | Echinenone (µg/g DW) | | | Canthaxanthin  (µg/g DW) | | | Phoenicoxanthin  (µg/g DW) | | | Astaxanthin (µg/g DW) | | | Phoenicoxanthin Ester (µg/g DW) | | |
| --- | --- | --- | --- | --- | --- | --- | --- | --- | --- | --- | --- | --- | --- | --- | --- | --- |
| MM | WT | n/d | | | n/d | | | n/d | | | n/d | | | n/d | | |
| MM:*Crt*ZW10-1 | Hemi | 0.4 | ± | 0.01 | 1.9 | ± | 0.1 | 9.0 | ± | 0.1 | 2.5 | ± | 1.0 | 2.4 | ± | 1.7 |
| MM:*Crt*ZW10-4 | Azy | n/d | | | n/d | | | n/d | | | n/d | | | n/d | | |
| MM:*Crt*ZW10-5 | Hom | n/d | | | n/d | | | n/d | | | n/d | | | n/d | | |
| MM:*Crt*ZW10-6 | Azy | n/d | | | n/d | | | n/d | | | n/d | | | n/d | | |
| MM:*Crt*ZW10-9 | Azy | n/d | | | n/d | | | n/d | | | n/d | | | n/d | | |
| MM:*Crt*ZW10-10 | Hom | n/d | | | n/d | | | n/d | | | n/d | | | n/d | | |
| MM:*Crt*ZW10-14 | Azy | n/d | | | n/d | | | n/d | | | n/d | | | n/d | | |
| MM:*Crt*ZW10-16 | Hemi | 6.9 | ± | 2.0 | 4.5 | ± | 0.7 | 23.1 | ± | 0.9 | 26.9 | ± | 3.2 | 8.1 | ± | 0.8 |
| MM:*Crt*ZW10-17 | Hemi | 3.4 | ± | 0.2 | 5.0 | ± | 0.7 | 18.1 | ± | 0.4 | 15.7 | ± | 2.2 | 7.3 | ± | 1.1 |
| MM:*Crt*ZW12-4 | Hemi | 2.1 | ± | 0.1 | 3.9 | ± | 0.7 | 24.0 | ± | 1.1 | 22.7 | ± | 2.6 | 7.0 | ± | 0.9 |
| MM:*Crt*ZW12-5 | Azy | n/d | | | n/d | | | n/d | | | n/d | | | n/d | | |
| MM:*Crt*ZW12-7 | Hemi | 1.1 | ± | 0.4 | 5.1 | ± | 1.5 | 16.9 | ± | 5.0 | 8.5 | ± | 4.8 | 5.4 | ± | 0.6 |
| MM:*Crt*ZW12-8 | Azy | n/d | | | n/d | | | n/d | | | n/d | | | n/d | | |
| MM:*Crt*ZW12-9 | Hemi | 0.9 | ± | 0.1 | 3.9 | ± | 0.6 | 15.5 | ± | 1.4 | 7.5 | ± | 0.6 | 5.4 | ± | 3.6 |
| MM:*Crt*ZW12-10 | Azy | n/d | | | n/d | | | n/d | | | n/d | | | n/d | | |
| MM:*Crt*ZW12-11 | Hemi | 1.4 | ± | 0.8 | 4.5 | ± | 1.4 | 15.0 | ± | 1.0 | 3.5 | ± | 1.1 | 9.1 | ± | 0.4 |
| MM:*Crt*ZW12-12 | Hemi | 2.4 | ± | 0.04 | 3.4 | ± | 0.2 | 21.2 | ± | 4.6 | 14.0 | ± | 3.7 | 7.6 | ± | 1.8 |
| MM:*Crt*ZW12-14 | Azy | n/d | | | n/d | | | n/d | | | n/d | | | n/d | | |

**Supplementary Table 3f.** Levels of novel ketocarotenoids in ripe fruit from T1 plants transformed with *Brevundimonas* sp. *crt*Z and *crt*W.

The values shown are the mean ± standard deviation, where n=3 to 10. n/d: Not detected; Zyg: zygosity; WT: wild type; Azy: azygous; Hemi: hemizygous; Hom: homozygous.

**Supplementary Table 4a.** Levels of endogenous leaf pigments in T2 plants transformed with *Brevundimonas* sp. *crt*Z and *crt*W.

| Line | Zyg | β-carotene (µg/g DW) | | | Lutein (µg/g DW) | | | Violaxanthin (µg/g DW) | | | Neoxanthin (µg/g DW) | | | Chlorophyll a (m/g DW) | | | Chlorophyll b (mg/g DW) | | |
| --- | --- | --- | --- | --- | --- | --- | --- | --- | --- | --- | --- | --- | --- | --- | --- | --- | --- | --- | --- |
| MM | WT | 616.0 | ± | 58.9 | 1125.3 | ± | 74.2 | 330.7 | ± | 34.5 | 50.0 | ± | 6.0 | 3.8 | ± | 0.24 | 1.5 | ± | 0.1 |
| 10-16-1 | Hemi | **42.8** | ± | **4.8***** | **292.0** | ± | **42.7***** | n/d | ± |  | n/d | ± |  | **2.6** | ± | **0.1***** | **0.1** | ± | **0.01***** |
| 10-16-2 | Hemi | **41.2** | ± | **2.7***** | **284.4** | ± | **9.4***** | n/d | ± |  | n/d | ± |  | **2.3** | ± | **0.03***** | **0.1** | ± | **0.007***** |
| 10-16-3 | Hemi | **47.7** | ± | **4.6***** | **352.1** | ± | **21.3***** | n/d | ± |  | n/d | ± |  | **2.6** | ± | **0.1***** | **0.1** | ± | **0.01***** |
| 10-16-5 | Azy | **660.1** | ± | **49.9*** | 1137.4 | ± | 70.8 | **239.5** | ± | **15.5***** | 113.2 | ± | 18.1 | 4.2 | ± | 0.3 | **1.9** | ± | **0.1*** |
| 10-16-6 | Azy | 675.8 | ± | 51.2 | 1098.8 | ± | 27.5 | **233.4** | ± | **16.5***** | 96.6 | ± | 3.1 | 3.6 | ± | 0.2 | 1.6 | ± | 0.1 |
| 10-16-15 | Azy | 592.8 | ± | 64.9 | 1054.3 | ± | 36.1 | **260.5** | ± | **9.3***** | 64.9 | ± | 29.4 | 3.5 | ± | 0.2 | 1.4 | ± | 0.2 |
| 10-16-8 | Hemi | **54.7** | ± | **12.8***** | **381.9** | ± | **40.1***** | n/d | ± |  | n/d | ± |  | **3.2** | ± | **0.2*** | **0.2** | ± | **0.04***** |
| 10-16-10 | Hemi | **48.7** | ± | **7.1***** | **376.4** | ± | **33.6***** | n/d | ± |  | n/d | ± |  | **3.1** | ± | **0.1***** | **0.1** | ± | **0.02***** |
| 10-16-13 | Hemi | **45.0** | ± | **18.7***** | **345.1** | ± | **9.6***** | n/d | ± |  | n/d | ± |  | **3.1** | ± | **0.03***** | **0.1** | ± | **0.05***** |
| 10-16-18 | Hemi | **39.5** | ± | **2.9***** | **342.9** | ± | **4.3***** | n/d | ± |  | n/d | ± |  | **2.9** | ± | **0.03***** | **0.1** | ± | **0.008***** |
| 10-16-19 | Hemi | **40.9** | ± | **3.5***** | **361.4** | ± | **16.4***** | n/d | ± |  | n/d | ± |  | **3.0** | ± | **0.1***** | **0.1** | ± | **0.01***** |
| 12-4-1 | Hom | **30.6** | ± | **0.2***** | **119.8** | ± | **0.8***** | n/d | ± |  | n/d | ± |  | **0.6** | ± | **0.003***** | **0.07** | ± | **0.0001***** |
| 12-4-3 | Hom | **27.8** | ± | **0.02***** | **85.5** | ± | **0.8***** | **8.0** | ± | **0.02***** | **1.4** | ± | **0.4***** | **0.3** | ± | **0.002***** | **0.07** | ± | **0.0003***** |
| 12-4-16 | Hom | **9.5** | ± | **0.2***** | **50.0** | ± | **0.2***** | n/d | ± |  | n/d | ± |  | **0.3** | ± | **0.002***** | **0.03** | ± | **0.0006***** |
| 12-4-21 | Hom | **3.4** | ± | **0.2***** | **15.6** | ± | **2.4***** | n/d | ± |  | n/d | ± |  | **0.07** | ± | **0.003***** | **0.009** | ± | **0.0005***** |
| 12-4-7 | Azy | 662.8 | ± | 65.7 | 1071.1 | ± | 18.2 | **289.7** | ± | **4.6**** | 55.7 | ± | 36.5 | 3.8 | ± | 0.1 | 1.6 | ± | 0.2 |
| 12-4-11 | Azy | **660.9** | ± | **32.1**** | 1209.6 | ± | 41.0 | **271.7** | ± | **9.6***** | **31.6** | ± | **0.8**** | **4.0** | ± | **0.08*** | **1.8** | ± | **0.09**** |
| 12-4-15 | Azy | 682.7 | ± | 51.6 | 1146.8 | ± | 22.7 | **244.3** | ± | **10.1***** | **36.4** | ± | **2.0*** | 3.7 | ± | 0.3 | 1.7 | ± | 0.1 |
| 12-4-4 | Hemi | **59.7** | ± | **9.6***** | **339.7** | ± | **5.2***** | n/d | ± |  | n/d | ± |  | **3.1** | ± | **0.1***** | **0.2** | ± | **0.03***** |
| 12-4-6 | Hemi | **58.0** | ± | **8.7***** | **341.0** | ± | **2.8***** | n/d | ± |  | n/d | ± |  | **3.2** | ± | **0.02***** | **0.2** | ± | **0.02***** |
| 12-4-10 | Hemi | **46.4** | ± | **6.4***** | **336.8** | ± | **16.9***** | n/d | ± |  | n/d | ± |  | **3.0** | ± | **0.05***** | **0.1** | ± | **0.02***** |
| 12-4-14 | Hom | **17.0** | ± | **0.8***** | **83.3** | ± | **17.6***** | n/d | ± |  | n/d | ± |  | **0.5** | ± | **0.003***** | **0.05** | ± | **0.002***** |

The values shown are the mean ± standard deviation, where n=3 to 10. Significant differences, shown in bold, between the mean of MM:*Crt*ZW lines and the MM control, for each compound were evaluated using the Student’s *t*-tests (*, P< 0.05 ; **, P< 0.01 ; ***, P<0.001). n/d: Not detected; Zyg: zygosity; WT: wild type; Azy: azygous; Hemi: hemizygous; Hom: homozygous.

**Supplementary Table 4b.** Levels of novel ketocarotenoids in leaf tissue from T2 plants transformed with *Brevundimonas* sp. *crt*Z and *crt*W.

| Line | Zyg | Echinonone (µg/g DW) | | | 3’-OH Echinonone (µg/g DW) | | | Canthaxanthin (µg/g DW) | | | Phoenicoxanthin (µg/g DW) | | | Astaxanthin (µg/g DW) | | |
| --- | --- | --- | --- | --- | --- | --- | --- | --- | --- | --- | --- | --- | --- | --- | --- | --- |
| MM | WT | n/d | | | n/d | | | n/d | | | n/d | | | n/d | | |
| 10-16-1 | Hemi | 163.1 | ± | 8.1 | 38.4 | ± | 17.4 | 531.8 | ± | 13.8 | 401.1 | ± | 16.4 | 85.9 | ± | 1.9 |
| 10-16-2 | Hemi | 137.5 | ± | 3.0 | 45.1 | ± | 6.5 | 517.3 | ± | 11.0 | 374.4 | ± | 15.6 | 72.3 | ± | 1.0 |
| 10-16-3 | Hemi | 165.3 | ± | 11.0 | 46.3 | ± | 18.6 | 561.7 | ± | 15.5 | 392.5 | ± | 16.1 | 100.4 | ± | 2.9 |
| 10-16-5 | Azy | n/d | | | n/d | | | n/d | | | n/d | | | n/d | | |
| 10-16-6 | Azy | n/d | | | n/d | | | n/d | | | n/d | | | n/d | | |
| 10-16-15 | Azy | n/d | | | n/d | | | n/d | | | n/d | | | n/d | | |
| 10-16-8 | Hemi | 155.0 | ± | 22.8 | 82.4 | ± | 9.4 | 893.1 | ± | 122.3 | 471.3 | ± | 91.0 | 81.4 | ± | 7.6 |
| 10-16-10 | Hemi | 144.8 | ± | 10.8 | 80.5 | ± | 5.8 | 837.4 | ± | 24.7 | 371.8 | ± | 12.5 | 67.2 | ± | 13.4 |
| 10-16-13 | Hemi | 165.1 | ± | 18.5 | 79.4 | ± | 7.8 | 850.0 | ± | 15.4 | 378.6 | ± | 7.7 | 65.2 | ± | 3.5 |
| 10-16-18 | Hemi | 128.4 | ± | 2.0 | 60.0 | ± | 2.1 | 718.2 | ± | 13.2 | 300.0 | ± | 5.1 | 65.1 | ± | 1.0 |
| 10-16-19 | Hemi | 141.1 | ± | 5.5 | 71.9 | ± | 2.8 | 751.4 | ± | 7.9 | 359.8 | ± | 13.6 | 73.3 | ± | 2.1 |
| 12-4-1 | Hom | 38.1 | ± | 0.03 | 11.5 | ± | 2.0 | 105.1 | ± | 0.2 | 99.9 | ± | 0.2 | 31.6 | ± | 0.6 |
| 12-4-3 | Hom | 14.3 | ± | 0.03 | n/d | | | 37.8 | ± | 0.6 | n/d | | | 22.1 | ± | 0.5 |
| 12-4-16 | Hom | 19.3 | ± | 0.4 | 5.9 | ± | 1.0 | 42.4 | ± | 1.0 | 19.5 | ± | 8.5 | 25.9 | ± | 2.8 |
| 12-4-21 | Hom | 3.2 | ± | 0.02 | n/d | | | 11.0 | ± | 0.2 | 9.6 | ± | 2.4 | 18.0 | ± | 0.9 |
| 12-4-7 | Azy | n/d | | | n/d | | | n/d | | | n/d | | | n/d | | |
| 12-4-11 | Azy | n/d | | | n/d | | | n/d | | | n/d | | | n/d | | |
| 12-4-15 | Azy | n/d | | | n/d | | | n/d | | | n/d | | | n/d | | |
| 12-4-4 | Hemi | 176.6 | ± | 16.7 | 74.4 | ± | 4.7 | 943.0 | ± | 38.8 | 411.72 | ± | 2.0 | 74.5 | ± | 3.0 |
| 12-4-6 | Hemi | 163.8 | ± | 13.8 | 82.6 | ± | 8.3 | 850.4 | ± | 28.7 | 412.0 | ± | 21.5 | 75.4 | ± | 2.3 |
| 12-4-10 | Hemi | 139.1 | ± | 10.1 | 68.4 | ± | 6.1 | 851.8 | ± | 23.0 | 411.4 | ± | 20.4 | 79.5 | ± | 3.0 |
| 12-4-14 | Hom | 33.6 | ± | 0.4 | 12.7 | ± | 0.1 | 104.0 | ± | 0.3 | 106.5 | ± | 1.9 | 37.0 | ± | 6.4 |

The values shown are the mean ± standard deviation, where n=3 to 10. n/d: Not detected; Zyg: zygosity; WT: wild type; Azy: azygous; Hemi: hemizygous; Hom: homozygous.

**Supplementary Table 4c.** Pigment levels across 5 stages of fruit development and ripening in T2 plants transformed with *Brevundimonas* sp. *crt*Z and *crt*W.

| (µg/g DW) | 25 dpa | | 39 dpa | | 49 dpa | | 56 dpa | | 66 dpa | |
| --- | --- | --- | --- | --- | --- | --- | --- | --- | --- | --- |
|  | Azy | Hemi | Azy | Hemi | Azy | Hemi | Azy | Hemi | Azy | Hemi |
| Phytoene | 16.12  ± 3.84 | 14.65  ± 1.35 | n/d | 14.99  ± 1.16 | 20.82  ± 3.01 | 15.42  ± 2.00 | 22.64  ± 4.13 | **16.68  ± 2.31*** | 20.60  ± 2.83 | 19.96  ± 3.37 |
|  |  |  |  |  |  |  |  |  |  |  |
| Phytofluene | n/d | n/d | n/d | n/d | 238.94  ± 16.06 | **60.90 ± 7.26***** | 227.85  ± 41.65 | **141.71  ± 7.00***** | 219.10  ± 40.89 | **437.05  ± 123.68**** |
|  |  |  |  |  |  |  |  |  |  |  |
| ζ-Carotene | n/d | n/d | n/d | n/d | 111.16  ± 6.90 | 98.79  ± 6.23 | 102.98  ± 8.15 | 102.97  ± 5.25 | 109.92  ± 11.82 | **133.89  ± 18.87**** |
|  |  |  |  |  |  |  |  |  |  |  |
| Lycopene | n/d | n/d | n/d | n/d | 1107.35  ± 18.36 | **141.33  ± 2.89**** | 1112.11  ± 41.82 | 1153.79  ± 44.68 | 1844.22  ± 216.67 | **3426.12  ± 1310***** |
|  |  |  |  |  |  |  |  |  |  |  |
| β-Carotene | 64.83  ± 5.06 | 58.37  ± 5.78 | 72.70  ± 1.85 | 59.18  ± 5.05 | 180.61  ± 15.21 | **61.36  ± 4.79**** | 168.89  ± 18.75 | **56.32  ± 2.71**** | 164.99  ± 17.07 | **63.45  ± 3.5**** |
|  |  |  |  |  |  |  |  |  |  |  |
| Lutein | 52.62  ± 5.19 | **4.60  ± 2.80***** | 38.56  ± 8.45 | **5.63  ± 2.07**** | 28.16  ± 1.94 | **2.42  ± 0.86***** | 20.33  ± 4.83 | n/d | 13.51  ± 2.89 | n/d |
|  |  |  |  |  |  |  |  |  |  |  |
| Violaxanthin | 60.80  ± 3.85 | n/d | n/d | n/d | n/d | n/d | n/d | n/d | n/d | n/d |
|  |  |  |  |  |  |  |  |  |  |  |
| Neoxanthin | 72.53  ± 5.11 | n/d | n/d | n/d | n/d | n/d | n/d | n/d | n/d | n/d |
|  |  |  |  |  |  |  |  |  |  |  |
| Chlorophyll a | 984.50  ± 183.54 | 1062.87  ± 645.56 | 785.83  ± 194.26 | 735.99  ± 136.52 | n/d | 430.41  ± 98.25 | n/d | n/d | n/d | n/d |
|  |  |  |  |  |  |  |  |  |  |  |
| Chlorophyll b | 250.51  ± 42.70 | **132.73  ± 32.20**** | 182.92  ± 49.38 | **93.00  ± 27.44*** | n/d | 49.73  ± 27.41 | n/d | n/d | n/d | n/d |
|  |  |  |  |  |  |  |  |  |  |  |
| Pheophytin | 1480.81  ± 447.46 | 883.62  ± 289.88 | 816.22  ± 76.68 | **478.65**  **± 113.70***** | 216.88  ± 30.91 | **2249.33  ± 873.85**** | 25.39  ± 2.17 | **51.41  ± 8.07*** | n/d | n/d |
|  |  |  |  |  |  |  |  |  |  |  |
| Echinenone | n/d | 0.96  ± 0.48 | n/d | 2.71  ± 1.42 | n/d | 1.52  ±0.55 | n/d | 4.20  ± 0.93 | n/d | 5.00  ± 1.97 |
|  |  |  |  |  |  |  |  |  |  |  |
|  |  |  |  |  |  |  |  |  |  |  |
| 3’OH-Echinenone | n/d | 0.42  ± 0.24 | n/d | 0.41  ± 0.16 | n/d | 1.62  ±0.26 | n/d | 2.06  ± 0.10 | n/d | 3.13  ± 1.34 |
|  |  |  |  |  |  |  |  |  |  |  |
| 3OH-Echinenone | n/d | n/d | n/d | n/d | n/d | 10.27 ±3.23 | n/d | 17.70  ± 3.58 | n/d | 6.34  ± 3.01 |
|  |  |  |  |  |  |  |  |  |  |  |
| Canthaxanthin | n/d | 6.00  ± 2.64 | n/d | 4.87  ± 2.29 | n/d | 15.76  ± 1.50 | n/d | 14.59  ± 0.93 | n/d | 15.48  ± 3.72 |
|  |  |  |  |  |  |  |  |  |  |  |
| Phoenicoxanthin | n/d | 1.51  ± 0.59 | n/d | 1.60  ± 0.59 | n/d | 2.17  ± 0.07 | n/d | 2.45  ± 0.10 | n/d | 3.06  ± 0.48 |
|  |  |  |  |  |  |  |  |  |  |  |
| Astaxanthin | n/d | 32.18  ± 11.21 | n/d | 46.06  ± 11.06 | n/d | 28.57  ± 1.55 | n/d | 42.83  ± 1.44 | n/d | 75.03  ± 15.24 |
|  |  |  |  |  |  |  |  |  |  |  |
| Total free ketos | n/d | 41.06  ± 14.30 | n/d | 55.64  ± 15.02 | n/d | 39.03  ± 17.82 | n/d | 83.83  ± 6.18 | n/d | 103.49 ± 11.61 |
|  |  |  |  |  |  |  |  |  |  |  |
| Phoenicoxanthin esters | n/d | 0.25  ± 0.04 | n/d | 1.60  ± 0.36 | n/d | 1.62  ± 0.39 | n/d | 1.03  ± 0..02 | n/d | 1.37  ± 0.16 |

The values shown are the mean ± standard deviation, where n=3 to 10. Significant differences, shown in bold, between the azygous control and the hemizygous line expressing *crt*Z and *crt*W, for each endogenous compound at each time point were evaluated using the Student’s *t*-tests (*, P< 0.05 ; **, P< 0.01 ; ***, P<0.001). dpa: Days post-anthesis; Zyg: zygosity; Azy: azygous; Hemi: hemizygous; n/d: not detected.

**Supplementary Table 5.** Determination of fruit softening across ripening in T2 plants transformed with Brevundimonas sp. *crt*Z and *crt*W.

|  | 39 dpa | | | 49 dpa | | | 56 dpa | | | 66 dpa | | |
| --- | --- | --- | --- | --- | --- | --- | --- | --- | --- | --- | --- | --- |
| MM azygous | 95.1 | ± | 2.2 | 65.8 | ± | 7.2 | 55.5 | ± | 3.5 | 49.8 | ± | 5.2 |
| MM:*Crt*ZW | **98.1** | ± | **1.2**** | **97.9** | ± | **3.3***** | **74.8** | ± | **1.7***** | **66.0** | ± | **7.8**** |

The values shown are expressed as a firmness percentage as determined using a Qualitest^TM^ firmness meter and are the mean ± standard deviation, where n=6 to 12. Significant differences, shown in bold, between the MM:*Crt*ZW and those of the azygous control at each developmental and ripening stage were evaluated using the Student’s *t*-tests (*, P< 0.05 ; **, P< 0.01 ; ***, P<0.001). dpa; days post anthesis.

**Supplementary Table 6.** Sequences of primers used in real-time RT-PCR and PCR.

| Gene ID | Accession number | Forward primer sequence | Reverse primer sequence |
| --- | --- | --- | --- |
| *DXS* | AF143812 | GCGGAGCTATTTCACATGGT | CTGCTGAGCATCCCAAT |
| *GGPPS1* | PQ267902 | GACAGCATCTGAGTCCGTCA | CTTGGCCAGGACAGAGTAGC |
| *GGPPS2* | SGN-U223568 | GGGATTGGAAAAGGCTAAGG | AGCAATCAATGGAGCAGCTT |
| *PSY1* | Y00521 | TGGCCCAAACGCATCATATA | CACCATCGAGCATGTCAAATG |
| *PSY*2 | L23424 | GTTGATGGCCCTAATGCATCA | TCAAGCATATCAAATGGCCG |
| *PDS* | X59948 | GTGCATTTTGATCATCGCATTGAAC | GCAAAGTCTCTCAGGATTACC |
| *ZDS* | AF195507 | TTGGAGCGTTCGAGGCAA T | AGAAATCTGCATCTGGCGTATAGA |
| *CRTISO* | AF416727 | TTTTGGCGGAATCAACTACC | GAAAGCTTCACTCCCACAGC |
| *ZISO* | AK326152.1 | GCTCACACGCTGTGGATTGGGAATTCAGTT | CGGTCCCCATTCCAGGCACC |
| *LCYB* | AF254793 | TCGTTGGAATCGGTGGTACAG | AGCTAGTGTCCTTGCCACCAT |
| *CYCB* | Y18297 | TGTTATTGAGGAAGAGAAATGTGTGAT | TCCCACCAATAGCCATAACATTTT |
| *LCYE* | Y14387 | AACACTTGCATTTGGTGCTG | AGTACAGAGGCGCATTTTGG |
| *CRTRB1* | Y14809 | CTCGAGGATGAGAAGCTGAAACCTC | GCCAAGCGAGTAGCTAAGATCTGTT |
| *ZEP* | Z83835.1 | TTGGGTTTTAGGAGGCAATG | CCCGCAGGTAAAAGTAACCA |
| *VDE* | NM_001247681 | CCCCTTGTTGAACGGTTAGA | GCAGCTCTTTGAAACCTTCG |
| *ACTIN* | BT013524 | AGGTATTGTGTTGGACTCTGGTGAT | ACGGAGAATGGCATGTGGAA |
| *NPTII* | U89673 | GGCGTTCCTTGCGCAGCT | CGTGCTCGCTCGATGATGCGA |
| *crtZ* | AB377272 | TGGGAATGGAGGCTTTCGCTTGG | ACGATAGCAGGAGCAGCGAAG |
| *crtW* | AB377271 | TCTGGGCTGCTCCTGCTCTT | AGCGTGGTGAGCATCAGCGA |
| *PDSg* | X78271 | CTAGGTTCTTGCTGCCTTGC | CCAACTTTTTGGCAATGCTT |

*DXS*, 1-deoxy-D-xylulose-5-pyrophosphate synthase. *GGPPS1*, geranylgeranyl pyrophosphate synthase-1. *GGPPS2,* geranylgeranyl pyrophosphate synthase-2. *PSY1,* phytoene synthase-1. *PSY2,* phytoene synthase-2. *PDS,* phytoene desaturase. *ZDS*, ζ-carotene desaturase. *CRTISO,* carotene isomerase*. ZISO, ζ-carotene isomerase. CYCB,* lycopene cyclase B. *LCYB*, β-lycopene cyclase. *LCYE,* ε-lycopene cyclase. CRTRB1, β-carotene hydroxylase. ZEP, zeaxnthin epoxidase. VDE, violaxanthin deepoxidase. *NPTII*, neomycin phosphotransferase II. *crtW*, synthetic ketolase based on the *Brevundimonas sp.* sequence. *crtZ,* synthetic hydroxylase based on the *Brevundimonas sp.* sequence. *PDSg,* phytoene desaturase genomic sequence.

**Supplementary Figure 1.** Thin layer chromatographic separation of pigments from MM and MM:*Crt*ZW leaf tissue. Lane 1: standards, lane 2: MM, lane 3: MM:*Crt*ZW6, lane 4: MM:*Crt*ZW7, lane 5: MM:*Crt*ZW10, lane 6: MM:*Crt*ZW12. a: echinenone, b: 3-hydroxyechinenone, c: canthaxanthin, d: phoenicoxanthin, e: astaxanthin, f: zeaxanthin, g: β-carotene.

5

6

4

1

2

3

g

f

e

d

c

b

a


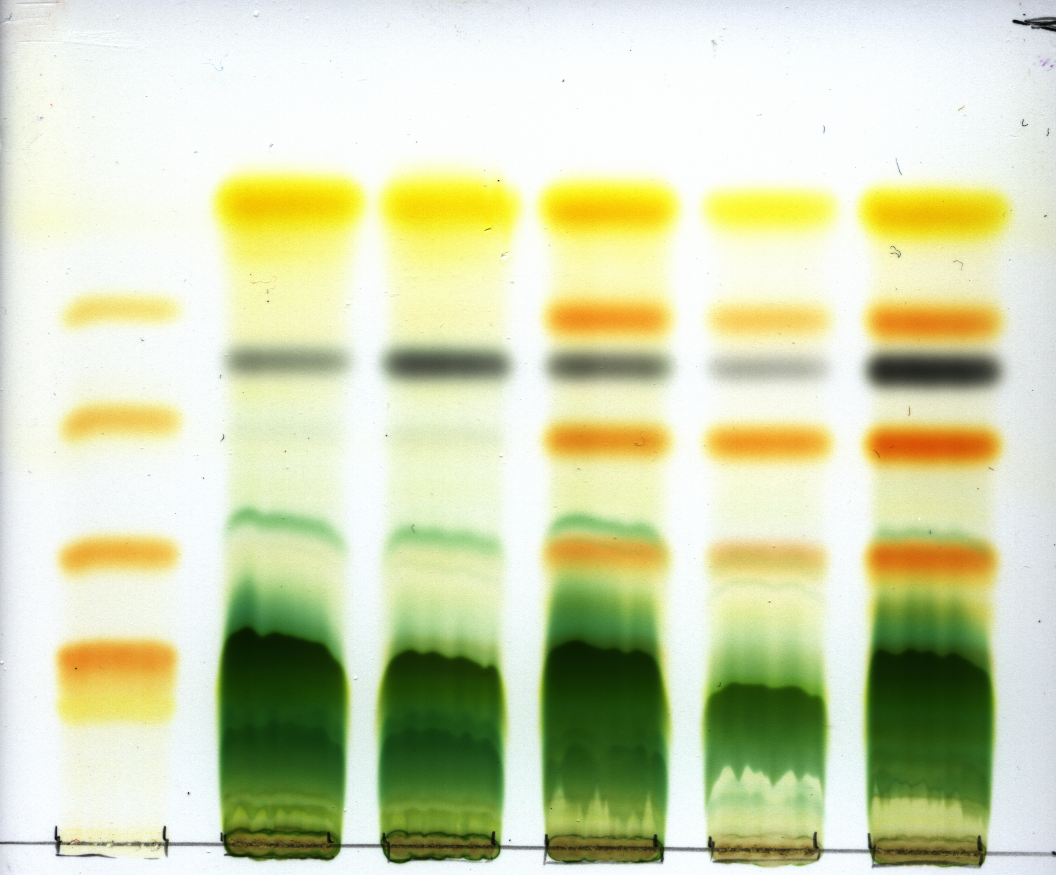


**Supplementary Figure 2.** Principal Component Analysis of leaf, mature green and ripe fruit tissues from MM:*Crt*ZW plants relative to the azygous control.


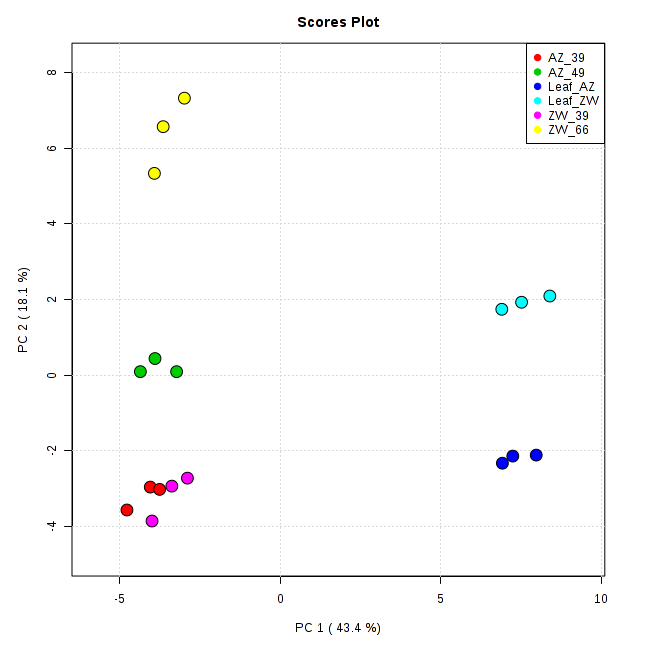


A minimum of three biological and three technical replicates were analysed and comparisons are based on ~70 metabolites identified in the polar extracts. Leaf_AZ (dark blue), azygous leaf tissue; Leaf_ZW (light blue) MM:*Crt*ZW leaf tissue; AZ_39 (red), mature green azygous fruit; ZW_39 (pink), mature green MM:*Crt*ZW fruit; AZ_49 (green), ripe azygous fruit; ZW_66 (yellow), ripe MM:*CrtI*ZW fruit.


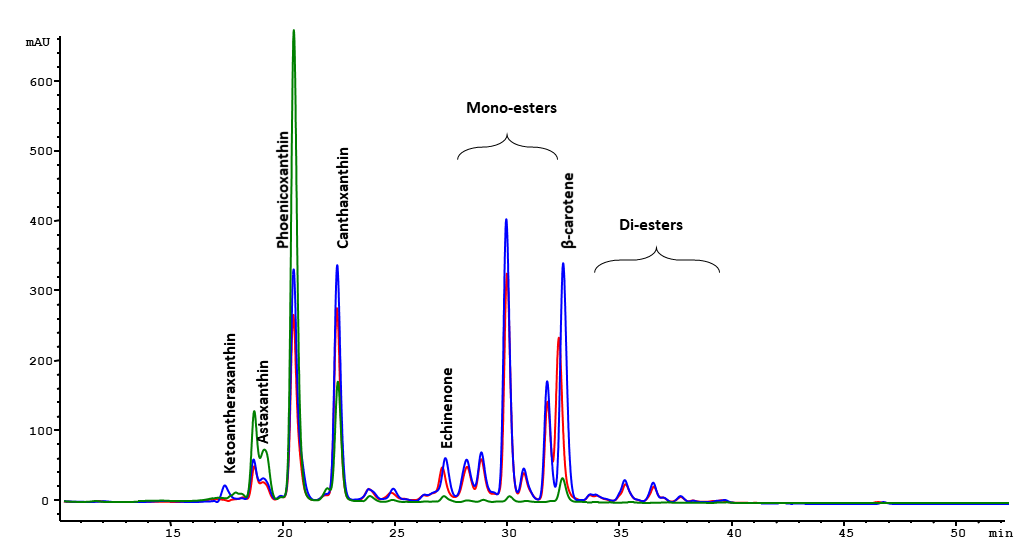


A.


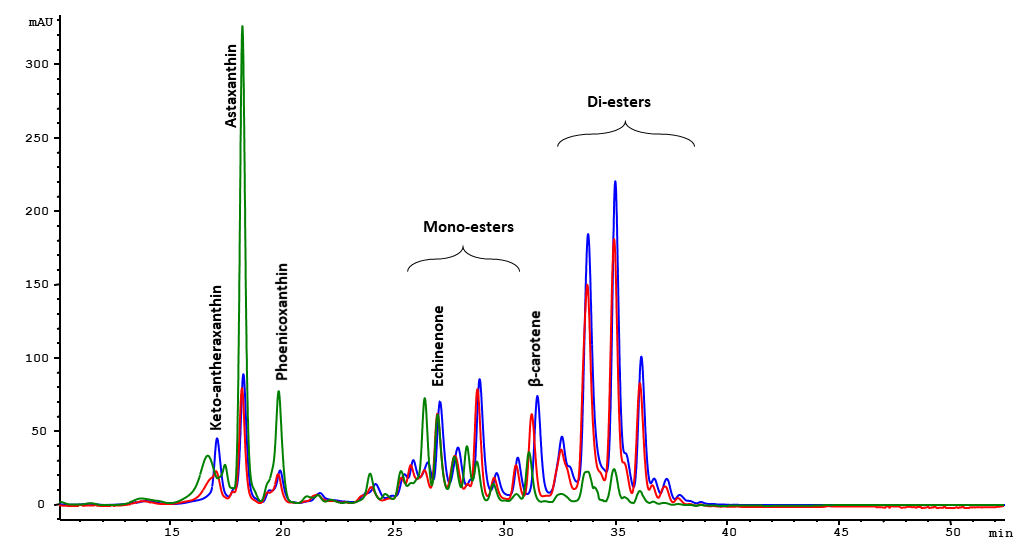


B..

**Supplementary Figure 3.** HPLC chromatograms at 470nm of ripe fruit extracts from a) HC/MM:*Crt*ZW and b) HU/MM:*Crt*ZW. Blue: extracts before enzymatic hydrolysis; Red: extracts incubated at 37^o^C for 45 min without cholesterol esterase; Green: extracts incubated at 37^o^C for 45 min with cholesterol esterase. A different manufacturer’s platform was used for this analysis resulting in altered retention times compared to those provided in Table S2. Carotenoids were identified by co-chromatography and comparison of spectral properties with authentic standards and reference spectra.


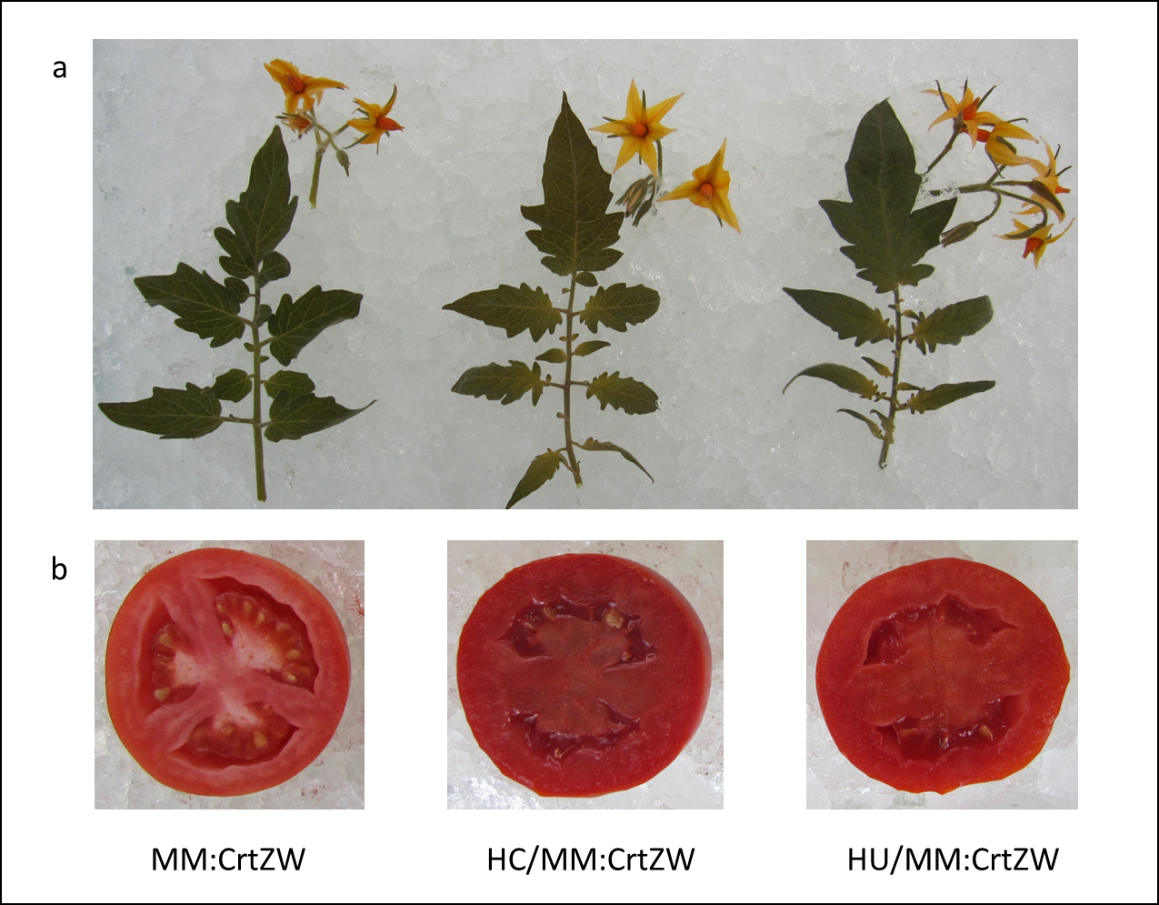


**Supplementary Figure 4.** Phenotypes of HC/MM:*Crt*ZW and HU/MM:*Crt*ZW compared to MM:*Crt*ZW.

a. Leaves and flowers; b. Ripe fruits.
